# Regulation of DNA Replication Timing on Human Chromosome by a Cell-Type Specific DNA Binding Protein SATB1

**DOI:** 10.1371/journal.pone.0042375

**Published:** 2012-08-07

**Authors:** Masako Oda, Yutaka Kanoh, Yoshihisa Watanabe, Hisao Masai

**Affiliations:** Genome Dynamics Project, Department of Genome Medicine, Tokyo Metropolitan Institute of Medical Science, Tokyo, Japan; Universita’ di Milano, Italy

## Abstract

**Background:**

Replication timing of metazoan DNA during S-phase may be determined by many factors including chromosome structures, nuclear positioning, patterns of histone modifications, and transcriptional activity. It may be determined by Mb-domain structures, termed as “replication domains”, and recent findings indicate that replication timing is under developmental and cell type-specific regulation.

**Methodology/Principal Findings:**

We examined replication timing on the human 5q23/31 3.5-Mb segment in T cells and non-T cells. We used two independent methods to determine replication timing. One is quantification of nascent replicating DNA in cell cycle-fractionated stage-specific S phase populations. The other is FISH analyses of replication foci. Although the locations of early- and late-replicating domains were common between the two cell lines, the timing transition region (TTR) between early and late domains were offset by 200-kb. We show that Special AT-rich sequence Binding protein 1 (SATB1), specifically expressed in T-cells, binds to the early domain immediately adjacent to TTR and delays the replication timing of the TTR. Measurement of the chromosome copy number along the TTR during synchronized S phase suggests that the fork movement may be slowed down by SATB1.

**Conclusions:**

Our results reveal a novel role of SATB1 in cell type-specific regulation of replication timing along the chromosome.

## Introduction

DNA replication occurs once and only once during S phase of cell cycle [1]. This strict regulation is achieved by multiple layers of mechanisms. Once DNA replication is initiated, three billion base-pair human genome is replicated in 7∼8 hrs [2]. Origins on the eukaryotic chromosomes can be classified according to the timing at which they are programmed to fire during S phase [3]. Studies in yeasts have established that intra-S checkpoint negatively regulates the firing of late or dormant origins [4]. This is also the case in mammalian cells as well [5]–[8]. Other factors including histone modifiers have also been shown to regulate the timing of origin firing [9]–[11]. On larger genomes of higher eukaryotes, replication is initiated from tens of thousands of loci on the genome [2]. It is known that clusters of origins are fired more or less simultaneously, and they may constitute distinct chromosome domains that may be referred to as “replication (timing) domain” [12]–[14]. The genome-wide landscape of replication timing domains were shown to change during the process of differentiation and different cell types may have distinct replication timing domain structures [12], [15]–[17]. As much as 50% of the genome may be regulated differentially in different cell types [16], and 20% of the mouse genome undergoes reorganization during development [12]. Replication timing is generally conserved between different species (e.g. between human and mice) [13]. Replication domains may be correlated with histone modification marks and most notably with chromosome proximity organization that can be estimated by 4C or high-C assays [13], [18], [19].

It would be important to identify factors that may be responsible for cell-type specific regulation of replication timing. We have been using the human chromosome 5 5q23.3/31.1 locus (will be referred to as 5q23/31 hereafter) containing a cytokine cluster region as a model locus for the study of replication program, since extensive studies have revealed the transcription factors and epigenetic regulation involved in cell-type-specific and simulation-dependent regulation of transcription in this region. We previously mapped an origin near the cytokine cluster and have shown that replication timing regulation is not affected by the activity of local origins [20]–[22].

We have compared replication timing of the 3.5 Mb segment of 5q23/31 in T cells and non-T cells, and found that the boundary of the early and late replication domain may be differentially regulated. Furthermore, a T cell specific-factor, SATB1 (Special AT-rich binding protein) [23]–[26], plays an important role in determination of the location of TTR (replication timing transition region). TTR forms a boundary between adjacent different timing domains, is often present near a synteny boundary, and is associated with higher frequency of SNP and increased occurrence of mutations that are often responsible for cancer [27]. Thus, elucidation of mechanisms for setting of TTR on chromosomes will also provide insight into the generation of mutation hot spots which may ultimately lead to diseases.

## Materials and Methods

### Cell Culture

Jurkat (Jurkat E6.1; Human T lymphocyte from acute T cell leukemia) and HL-60 (Human promyelocytic leukemia cells) were cultured in RPMI1640 (Nissui) supplemented with 10% fetal bovine serum (FBS), sodium bicarbonate, 3.5 mM L-glutamine, 2-mercaptethanol and antibiotics (100 U/ml Penicillin and 100 µg/ml Streptomycin (GIBCO)). HeLa S3 (Human epithelial carcinoma cell line) were cultured in Dulbecco’s modified Eagle’s medium (DMEM) supplemented with 10% FBS, sodium bicarbonate and 5 mM L-glutamine. All the cells were cultured in a humidified atmosphere containing 5% CO_2_. Jurkat and HL-60 were purchased from ATCC, and HeLa S3 was purchased from the Health Science Research Resources Bank (Tokyo, Japan).

### Analyses of Replication Timing

Replication timing analyses were performed as described previously [20], [28], [29]. Briefly, exponentially growing cells were pulse-labeled with 50 µM BrdU (5-bromo-2’-deoxyuridine; Roche) for 30 min, fixed in 70% ethanol, stained with propidium iodide (PI) and fractionated by fluorescence assisted cell sorting (FACS Vantage, Becton & Dickinson) into six (G1, S1–S4 and G2) cell cycle fractions, each containing 40,000 cells. After isolation of total genomic DNA, the DNA was sheared by sonication to the average size of 500 bp, denatured and newly replicated, BrdU-labeled DNA was immunoprecipitated using anti-BrdU antibody (BD). To determine the levels of target sequences in each fraction, semi-quantitative PCR was conducted using primers for individual loci and control loci reported previously (**Table S1** or primer sequences available on request). The band intensity of the BrdU-labeled target loci was quantified and was normalized by that of the BrdU-labeled mitochondorial DNA (mtDNA) in each cell cycle fraction [30]. PCR products were visualized on 8% acrylamide gels stained with SYBR Green I (Molecular probes). The obtained gel images were captured and band intensities were determined by using LAS-1000 (Fujifilm). Taq polymerase (Sigma-Aldrich or Genescript) or platinum Taq polymerase (Invitrogen) was used for PCR quantification of mtDNA or the genomic target DNA, respectively.

### FISH Analyses

For preparation of nuclei, cells, labeled with 30 µM BrdU for 10 min prior to harvest, were treated with 60 mM (Jurkat) or 75 mM (HL-60 and HeLaS3) potassium chloride (hypotonic condition) for 30–40 min at 37°C and fixed in three changes of ice-cold methanol: acetic acid (3∶1). Fixed cells were then dropped onto slides and dried at 55–60°C overnight. To denature chromosomal DNA, slides were preheated at 70°C on a heat block, placed in 70% formamide in 2X SSC at 72°C for 2 min, then transferred quickly to ice-cold 70% ethanol for 5 min, then to 90 and 100% ethanol for 5 min each, and air-dried.

BAC clones for the human chromosome 5 were purchased from Invitrogen and were used as probes. The position of each probe is shown in figures. The cosmid clone of cCl12–140 for the human chromosome 11 was kindly provided by Dr. K. Okumura [31]. Biotin-16-dUTP labeled probes were prepared by nick translation.

For each slide, 96 ng labeled probe was ethanol-precipitated in the presence of 4 µg human Cot-1 DNA (Roche) and 8 µg salmon sperm DNA (Nacalai tesque) as carrier. DNA was resuspended in 20 µl of hybridization buffer (50% formamide (v/v) and 10% dextran sulfate in 2X SSC), denatured at 80°C for 10 min, and preannealed at 37°C for 20–30 min before applying to the slide. The hybridization probe mixture was applied to each slide under a coverslip, and sealed with rubber solution. Each slide was incubated overnight at 37°C in a moist chamber, and was then washed three times with 50% formamide in 2X SSC for 5 min at 42°C, followed by three washes in 0.8X SSC for 5 min at 60°C. The slides were then incubated for 30 min in a blocking solution (3% bovine serum albumin fraction V (Sigma) and 0.1% Tween20 in 4X SSC) at 37°C and further incubated with anti-BrdU Alexa Fluor 546 conjugate (Invitrogen), Streptoavidin Alexa Fluor 488 conjugate (Invitrogen) and DAPI in a detection solution (1% BSA and 0.1% Tween20 in PBS) for 30 min at 37°C. Then slides were washed three times with PBST (0.1% Tween20 in PBS) for 5 min at 42°C, mounted in an antifade solution (23.3 mg/ml of DABCO (Sigma) and 20 mM Tris-HCl in 90% glycerol) and sealed with nail polish. Slides were stored at 4°C in the dark until observation.

To count the numbers of signals, hybridized slides were examined using 63Xoil objective on a Zeiss Axiophot fluorescence microscope fitted with a filter set for DAPI, Alexa Fluor 546 and Alexa Fluor 488. At least 200 S-phase nuclei were scored, and the signal patterns were classified as singlet-singlet (SS)/singlet-doublet (SD)/doublet-doublet (DD) or single (S)/double (D) in some cases.

### Gene Expression Analyses by RT-PCR

Jurkat and HL-60 were stimulated with 20 nM phorbol myristate acetate (PMA) and 1 µM ionomysin for 6 hrs. Total RNA (5 µg) was extracted with Trizol (Invitrogen) according to the manufacturer’s protocol and then reverse transcribed using a oligo(dT)_12–18_ primer and Expand Reverse Transcriptase (Roche). To prepare non-coding transcripts, total RNA (4 µg) was reverse transcribed using random primers (Takara).

PCR amplification of the genes of interest was carried out using the first-strand cDNA. The PCR products were visualized on agarose gel stained with SYBR Green I (Molecular probes). Gel images were captured and band intensities, determined by LAS-1000 (Fujifilm), were normalized to that of a housekeeping gene, GAPDH. Primer sequences and PCR condition for each primer set are available on request. The list of the genes examined are in **Table S3**.

### Genome Analyses through Bioinformatics

The genes used in the analysis were taken from NCBI H. sapiens Genome (http://www.ncbi.nlm.nih.gov/). Transposable elements were extracted from the 129.0–132.5 Mb segment of the human chromosome 5 by RepeatMasker analysis (http://www.repeatmasker.org/).

### Western Blotting and Antibodies

Immunoblotting was performed according to standard procedures. Protein samples were mixed with 5X sample buffer (0.25 M Tris-HCl (pH6.8), 10% SDS, 50% glycerol, 11% 2-mercaptoethanol, and 0.02% bromophenol blue), boiled, and separated on SDS-PAGE (7.5 to 10% acrylamide). Antibodies from commercial sources were as follows: SATB1 (ab49061, Abcom; #61182, BD Biosciences), α-tubulin (T5168, Sigma), peroxide-conjugated anti-rabbit IgG and anti-mouse IgG (711-036-152 and 711-036-151, respectively, from Jackson ImmunoResearch Laboratories, Inc.).

### Plasmid Construction

Poly−A(+) mRNA of Jurkat was prepared and reverse transcribed as described above. Full-length SATB1 cDNA was amplified by PCR using the following primers; huSATB1-N 5′-CCG CTC GAG ATG GAT CAT TTG AAC GAG GCA ACT-3′ and huSATB1-C 5′-GCT CTA GAT CAG TCT TTC AAA TCA GTA TTA AT-3′. PCR-products, digested with *Xba*I and *Xho*I, were gel-eluted and the resulting DNA fragment was inserted at the *Xba*I-*Xho*I sites of pCSII-EF-mKO2-Cdt1Δ*Xho*I (in which one *Xho*I to the 5′ of mKO2 was deleted; mKO2, monomeric Kusabira Orange 2; [32], generating pCSII-EF-mKO2Δ*Xho*I-SATB1. pCSII-EF-mKO2-Cdt1Δ*Xho*I digested by *Xba*I/*Xho*I was treated with the Klenow fragment and religated, generating a control vector pCSII-EF-mKO2Δ*Xho*I. The final clones were verified by sequencing.

### Transfection and Sorting of Cells Expressing MKO2-SATB1 or MKO2

HeLaS3 cells, plated at a density of 4×10^6^ cells per 10-cm plate, were incubated for 24–30 hr prior to transfection. The plasmids encoding mKO2 alone (pCSII-EF-mKO2Δ*Xho*I) or mKO2 fused to SATB1 (pCSII-mKO2Δ*Xho*I-SATB1) (10 µg each) was introduced into HeLaS3 cells using FuGENE HD Transfection Reagent (Roche) according to manufacturer’s protocol. After 24–48 hrs, cells were observed by Biozero BZ-8000 fluorescence microscope (Keyence, Inc.) to examine transfection efficiency. Then, the cells were labeled with 30 µM BrdU for 10 min prior to harvesting. Cells were collected in PBS containing of 2 µg/ml propidium iodide (SIGMA). Living cells expressing mKO2 were sorted by FACS Aria (BD Biosciences). The sorted mKO2-positive cells were used for replication timing analysis by FISH as described above.

### Short Hairpin RNA (shRNA)-mediated Knockdown

pRS vector or pRS-sh*SATB*1 (ORIGENE) was introduced into Jurkat cells (5×10^6^) by electroporation using Nucleofector (AMAXA). The sequences of *SATB*1-shRNAs are pRS-*SATB*1-shRNA-1, 5′-TCATCAAGTTCTTTCAGAACCAGCGGTAC-3′ and pRS-*SATB*1-shRNA-2, 5′-CAACACAGAGGTGTCTTCCGAAATCTACC-3′. Cells were incubated for 72 hrs and an aliquot of the transfected cells (5×10^5^) was used for western analysis to check the repression of expression. The rest of the cells were used for replication timing analyses by FISH as described above.

### Generation of Stable Cell Lines

The pCSII-EF-mKO2Δ*Xho*I or pCSII-mKO2Δ*Xho*I-SATB1 plasmid was transfected with the packaging plasmid (pCAG-HIVgp) and the VSV-G/Rev-expressing plasmid (pCMV-VSV-G-RSV-Rev) into 293T cells [32] using TransIT293 (Mirus). High-titer viral solutions were collected at 3 days after tansfection, and used for transduction into HeLaS3 cells. At 10 days after transduction, mKO2-positive clones were selected under Biozero BZ-8000 fluorescence microscope (Keyence, Inc.) and HeLaS3 cells expressing mKO2 or mKO2-SATB1 were established and stored. Expression of expected polypeptides was confirmed by Western blot analysis.

### Chromatin Immunoprecipitation (ChIP)

ChIP analysis was carried out in HeLaS3 cells and HeLaS3 expressing SATB1. In brief, cells at about 80% confluency (∼1×10^7^ cells) were cross-linked for 4 min at 37°C by addition of 1% formaldehyde, and 125 mM glycine was added to terminate the reaction. The cells were washed with ice-cold phosphate-buffered saline (PBS), harvested in buffer I (50 mM Hepes-KOH (pH 7.5), 140 mM NaCl, 1 mM EDTA, 10% glycerol, 0.5% NP-40, 0.2 5% Triton X-100, protease inhibitors and 1 mM phenylmethylsulfonyl fluoride (PMSF)), and incubated at 4°C for 10 min. After centrifugation, pellets were resuspended in lysis buffer II (10 mM Tris-HCl (pH 8.0), 200 mM NaCl, 1 mM EDTA, and 0.5 mM EGTA) and incubated at 4°C for 10 min. After centrifugation, pellets were resuspended in lysis buffer III (50 mM Hepes-KOH (pH 7.5), 140 mM NaCl, 1 mM EDTA, 0.5 mM EGTA, 1% TritonX-100, 0.1% sodium deoxycholate, and 0.1% SDS containing protease inhibitors and 1 mM PMSF) and sonicated in an ice-water bath until cross-linked chromatin DNA was sheared to an average length around 500 bp. Insoluble materials were removed by centrifugation at 15,000 rpm in a microcentrifuge at 4°C for 5 min. The sonicated cell supernatant was incubated with 10 µl of Protein A Sepharose beads (GE Healthcare) bound with 32 µg of salmon sperm DNA and 2 µg of mouse SATB1 antibody or mouse IgG1 at 4°C overnight. The beads were harvested by centrifugation and washed six times with RIPA wash buffer (50 mM HEPES-KOH (pH 7.0), 500 mM LiCl, 1 mM EDTA, 0.7% sodium deoxycholate, 1% NP-40, protease inhibitors and 1 mM PMSF) and once with TE (10 mM Tris-HCl (pH 8.0) and 1 mM EDTA). Chromatin antibody complexes were eluted from the beads by addition of elution buffer (50 mM Tris-HCl (pH 8.0), 10 mM EDTA, and 1% SDS). Cross-linking was reversed by incubation at 65°C overnight in TE containing 1%SDS. Protein K digestion was performed for 4 hrs at 37°C in digestion buffer (10 mM Tris-HCl (pH 8.0), 1 mM EDTA, 200 mM NaCl, 0.5 mg/ml of proteinase K, and 0.5 mg/ml of glycogen). ChIP DNA was precipitated with ethanol and dissolved in TE containing RNase A. After incubation for 1 hr at 37°C, ChIP DNA was purified by MinElute (QIAGEN) and used for quantitative PCR.

### Quantitative Real-time PCR Analysis

Real-time PCR was conducted using SYBR Premix EX Taq (Takara) with LightCycler480 (Roche Diagnostics). Primer sequences are available upon request. The PCR products were measured by SYBR green fluorescence.

### Cell Cycle Synchronization

Cell cycle synchronization was conducted as previously described [33]. Briefly, cells were arrested at the G1/S boundary by incubation in the presence of 2.5 mM thymidine for 16 hrs twice with a 9-hr interval of growth without thymidine. Arrested cells were released into cell cycle and harvested at the indicated times.

### Preparation and Purification of Genomic DNA

5×10^6^ cells were resuspended in 600 µl of lysis buffer (10 mM Tris-HCl (pH 8.0), 100 mM NaCl and 1 mM EDTA), and 30 µl of 10% SDS and 6 µl of 20 mg/ml Proteinase K was added, followed by gentle mix and incubation at 55°C for several hrs to overnight with rotation. After adding 0.6 ml of phenol/chloroform/isoamyl alcohol, the samples were mixed and centrifuged at 15,000 rpm for 5 min. The supernatant was treated with an equal volume of chloroform/isoamyl alcohol twice and DNA was precipitated by adding 1/10 volume of 3 M NaOAc and an equal volume of isopropanol. DNA was dissolved in 0.1 ml EB buffer (10 mM Tris-Cl (pH 8.5), QIAGEN). For quantification of the amount of genomic DNA at a particular locus, qPCR was conducted on a diluted set of the purified DNA solution at each time point after release from double thymidine block.

## Results

### Replication Timing of the 5q23/31 Locus on the Human Chromosome 5

In order to analyze regulation of replication timing in different cell types, we examined replication timing of a segment on the human chromosome 5 by quantification of the nascent DNA in S-phase stage-specific cell populations of Jurkat (Human T lymphocyte from acute T cell leukemia) and HL-60 (Human promyelocytic leukemia cells, non-T lymphocyte), which are different types of mature leukemia cells. We focused on the 3.5-Mb segment of 5q23/31 containing clusters of cytokine genes whose transcription is regulated in a T cell-type specific manner [34]–[37].

**Figure 1:**
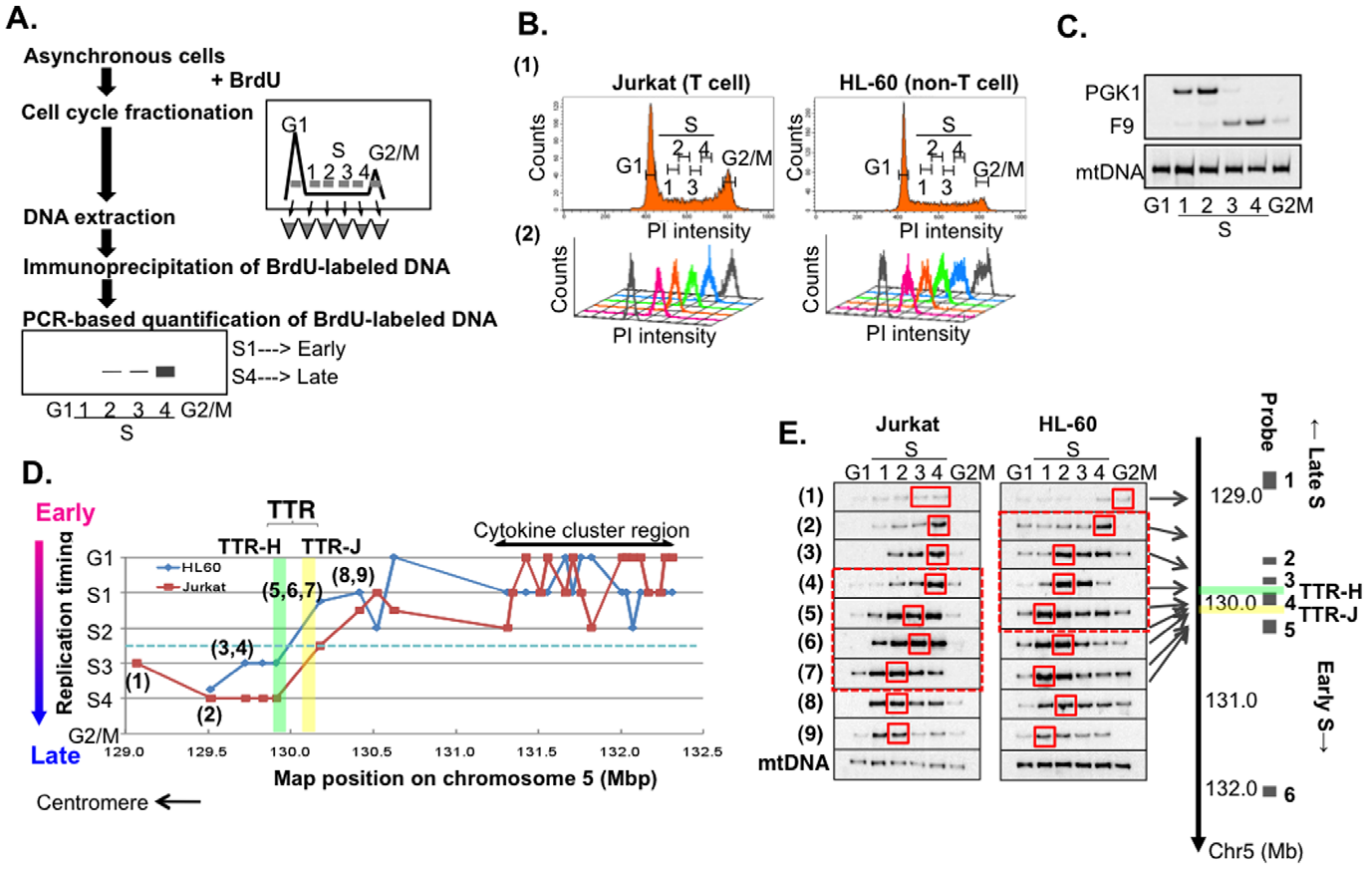
Replication timing of the human 5q23/31. **A.** Experimental strategy for determination of replication timing. Asynchronously replicating cells were labeled with BrdU and sorted by FACS into six fractions (G1, S1–4, G2/M) on the basis of DNA content. Genomic DNA from cells in each fraction was extracted, and newly replicated DNA was immunoprecipitated with anti-BrdU antibody. Semi-quantitative PCR was carried out using the newly replicated DNA as template. Relative band intensity was quantified. The values in each fraction were normalized by the levels of BrdU-labeled mitochondrial DNA (mtDNA; replicated equally throughout the cell cycle) used as an internal control for the recovery of DNA in each sample. **B.** 40,000 cells (Jurkat and HL-60) sorted (upper) and collected on the basis of DNA content (G1, S1–4, G2/M) were stained with PI, and analyzed by FACS (lower). **C.** Validation of cell cycle fractionation. The known early (PGK1) or late (F9) replicating region is enriched in appropriate fractions in comparison with the level of mtDNA in HL-60. **D.** DNA replication timing on the human chromosome 5q23/31 region (3.5 Mb) containing the cytokine cluster region in Jurkat (T cell) and HL-60 (non T cell). The 2.2 Mb segment containing the cytokine cluster (130.3–132.5) replicates in G1 or early in the S-phase (S1 and S2), whereas the 0.9 Mb segment distal to the cluster and proximal to the centromere replicates late in the S phase (S3, S4 and G2). The mean locations of the timing transition region (TTR) are located at around 130.15–130.25 (yellow box) in Jurkat (TTR-J) and at around 129.95–130.05 (green box) in HL-60 (TTR-H), and are offset by 180 kb in the two cell types. The left boundary of early replicating region coincided with the transition of chromosomal synteny (see **Fig. S2A**). **E.** The results of replication timing assays with fractionated cells are shown for each location along the 3.5 Mb human chromosome. The locations of the 9 primers used are indicated along the 5q23/31 region shown to the right of the panels. Small red solid boxes show the peak timing fraction for each probe, and large red dotted boxes show the maximum timing transition segments for Jurkat (129.98–130.22) and HL-60 (129.52–130.16).

Nascent DNAs were labeled with BrdU, which is incorporated into newly replicated DNA in place of thymidine, and then we sorted the labeled cells into six fractions (representing G1, S2, S2, S3, S4, and G2/M cell cycle stages) by a cell sorter. Fractionation into expected cell cycle stage-specific populations was confirmed by FACS analyses (**Fig. 1A and B**). The BrdU-labeled DNA was immunoprecipitated and enrichment of newly replicated DNA in each fraction was analyzed by PCR-based assays [20], [28], [29]. The values were corrected by the level of BrdU-labeled mtDNA which replicates equally throughout the cell cycle (**Fig. 1C**) [30]. The fraction containing the highest level of BrdU-labeled DNA indicated the replication timing. As positive control, we first analyzed replication timing of PGK1 (phosphoglycerate kinase I) and F9 (coagulation factor IX) known to be replicated in early and late S phase, respectively [28], [38]. As expected, PGK1 and F9 replicated in the early and late S phase, respectively, in HL60 cells (**Fig. 1C**). We noted that the distribution of replication timing is generally broader and show variations at different locations in TTR. This may suggest a possibility that the precise timing may vary in different single cells or in two alleles, and this variation may be larger in TTR compared to other regions. This could be due to the variation of the fork speed when it proceeds in TTR.

We then examined the replication timing of the 3.5-Mb segment at 5q23/31 by using locus-specific primers. We determined the S phase stages showing the highest enrichment of BrdU-labeled DNA, which represented the time of replication, and aligned the data along the segment at 5q23/31 (**Fig. 1D** **and Table S1**). The segment containing the cytokine cluster (130.3–132.5 Mb on chr5) replicates early (G1–S2) in both cells, in spite of differences in cell-types and gene expression profiles. On the other hand, the adjacent segment (129.0–129.9 Mb on chr5) replicates late (S3, S4) in both cell types (**Fig. 1D**). Thus, early- and late-replicating domains are basically conserved on the 3.5-Mb segment. A replication timing transition region (TTR) is present between the early- and late-replicating domains. The detailed analyses of this region revealed that TTR in two cell types are offset by about 200 kb. This suggests cell type-specific mechanisms may be involved in determination of TTR.

### Cell-type Specific Replication Timing Transition Region

In order to more precisely determine TTR in HL-60 and Jurkat, we analyzed replication timing in more details. The locations of timing boundary are offset by 180-kb in the two cell types (**Fig. 1D**); they are located between the coordinates 129.52 and130.16 Mbp in HL-60 (non-T cell) and between 129.98–130.22 in Jurkat (T cell) (**Fig. 1E** and **Fig. S1**). On the basis of the locations that give a mid-S timing (a blue dotted line in **Fig. 1D**), we have tentatively defined the segments at 129.95–130.05 Mb or 130.15–130.25 Mb as TTR-H or TTR-J, respectively.

**Figure 2:**
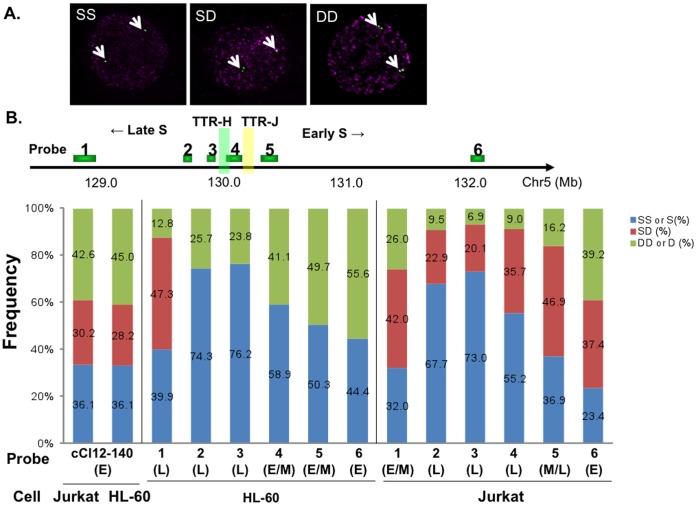
Analyses of replication timing by FISH. **A.** Hybridization signals of replicating cells. SS, singlet-singlet: SD, singlet-doublet; DD, doublet-doublet. **B.** Replication timing analyzed by FISH at 5q23/31. Locations of DNA probes derived from 5q23/31used in this study are shown at the top. Human BAC clones were purchased from Invitrogen. A cosmid clone on the chromosome 12, cCl12–140, was kindly provided by Dr. Okumura (Nogami et al, 2000), and was used as a control for early replication. At least 200 BrdU-positive nuclei (S-phase) were counted for each probe. The signal patterns were classified into SS, SD, or DD. On the haploid segment of HL-60 (probes 2∼6), two signal patterns, singlet (S) and doublet (D), were observed. Replication timing of the human 5q23/31, estimated from the FISH analyses, is consistent with that of the cell cycle fractionation studies (see text for details). E, E/M M/L and L stand for early-, early/mid-, mid/late- or late-replicating, respectively.

We next conducted FISH analyses to investigate replication timing at this locus (**Fig. 2A**). We counted the fluorescence signals in at least 200 S-phase nuclei labeled with BrdU in both cell types. The fluorescence signals were divided into three categories, two unreplicated dots (single-single, SS), one unreplicated and one replicated dot (single-double, SD) and two replicated dots (double-double, DD). By using the cCl12-140 probe representing an early replicating region in HL-60 [31], we confirmed early replication patterns at this locus in both HL-60 and Jurkat (**Fig. 2B**; DD, 45.0%; SD, 28.2%; SS, 36.1% in HL-60 and DD, 42.6%; SD, 30.2%; SS, 36.1% in Jurkat). We then conducted FISH analyses using probes at the human chromosome 5q23/31 (**Table S2 and** **Fig. 2B**). The fluorescence signals with the probes 2∼6 in HL-60 only gave single (S, unreplicated) or double (D, replicated) dots, indicating that this region in HL-60 is haploid. FISH analyses can distinguish replicated and unreplicated chromosomes on both diploid and haploid genome regions, and we set the threshold for early replication in diploid or haploid locus at the DD or D value of approximately 40% that is larger that the value for SS or S, respectively.

With the probe 6, the frequency of ”replicated” signals was higher than that of “unreplicated” signals both in Jurkat (DD, 39.2%>SS, 23.4%) and HL-60 (D, 55.6%>S, 44.4%), indicating that this segment is early-replicating in both cells. With the probe 1, the frequency of “unreplicated” signals was higher than that of “replicated” signals both in HL-60 (DD, 12.8%<SS, 39.9%), indicating that this is late-replicating in HL-60 cells. It should be noted that probe 1 locus is replicated at mid/early in Jurkat (DD, 26.0%<SS, 32.0%) and at early in HeLaS cells. The mechanisms behind this sort of localized difference of replication timing among different cell types are not known. With the probes 4 in HL-60, the frequency of D sharply increased (23.8%−>41.1%) and the frequency of S decreased (76.2%−>58.9%) compared to the probe 3 (**Fig. 2B**). These data indicated that the location between the probes 3 and 4 marks the timing transition in HL-60. This conclusion is consistent with that determined by semi-qPCR of the timing-specific nascent DNA (**Fig. 1**). On the other hand, in Jurkat, DD/SS at the probe 4 is 9.0%/55.2%, whereas that at the probe 5 is 16.2%/36.9%, The values become 39.2%/23.4% at the probe 6, indicating that the location between the probes 4 and 6 marks the timing transition in Jurkat. This result is consistent with TTR determined by semi-qPCR of the timing-specific nascent DNA in Jurkat (**Fig. 1**). Thus, these data further support the presence of the cell type specific TTR.

Although replication timing domains are generally conserved among different species (e.g. between human and mouse) [13], cell-type specific replication timing may be observed in as much as 50% of the human genome [16]. Cell type-specific TTR may contribute to generation of some of these variations in replication timing domains.

### AT-rich Nature and the Absence of Coding Regions Characterize TTR

In order to understand the features of TTR, we analyzed distribution of genome composition, GC-content (%), gene density, and numbers of transposable elements (**Fig. S2**). Adjacent to the TTR, a synteny break point is present in rat and mouse. as is often found in other TTR (**Fig. S2A**; [27]. The early replicating region spanning the cytokine cluster segment is rich in GC content (>43%), as reported previously (**Fig. S2B**) [38], [39]. In contrast, the late replicating region has lower GC content (<43%). Analyses with narrower window ranges (10–100 kb) indicate that TTR is located in the segment with a lower GC content (AT-rich).

Although LINEs (long interspersed nuclear element) are rather uniformly scattered in both early- and late-replicating regions, higher numbers of LINEs (∼30 LINEs) are located near cytokine-cluster segment (**Fig. S2C**, shown by a black arrow). The highest density of LINEs, notably L1, is located within the TTR (32 LINE1s in the 25 kb segment, 130.025–130.05 Mb; shown by a red arrow). The frequency of SINEs (short interspersed nuclear element) is generally higher in early replicating regions (**Fig. S2D**). Another feature of TTR is the absence of genes (**Fig. S2E**). These findings suggest that TTR may reside in the AT-rich genome segment lacking genes and containing clusters of LINE1.

### Multiple Potential SATB1 Binding Sequences are Present Near TTR

Informatics examination of the 3.5-Mb segment of 5q23/31 revealed the presence of potential SATB1 (Special AT-rich sequence Binding protein 1) binding sites in the vicinity of the TTR. SATB1 binds to AT-rich regions and matrix attachment region (MAR). SATB1 binding sites were reported in previous studies and referred to as SBSs [40], [41]. Among the sixteen types of SBSs identified in Jurkat [40], sequences similar to the three types of SBSs (SBS-2, 4 and 14) were found on the 3.5 Mb segment (**Fig. S3A**), while those related to other types of SBSs (SBS-1, 3, 5∼13, 15 and 16) were not identified. SBS-2, 4 and 14 were located on the LINE1 sequence, especially on the L1P subfamily (**Table S4**). Furthermore, we searched the sequences with higher identity (≧85%) and bit scores (≧400), leading to “highly similar” SBSs (0 sequence for SBS-2, 9 sequences for SBS-4 and 39 sequences of SBS-14; **Fig. S3B**). Interestingly, these “highly similar” SBSs were located and clustered close to TTR (**Fig. S3C**). Since SATB1 is specifically expressed in T-cells, the results suggested a possibility that SATB1 may be involved in generation of cell-type specific TTR.

### SATB1 Expression Correlates with Replication Timing at TTR

**Figure 3:**
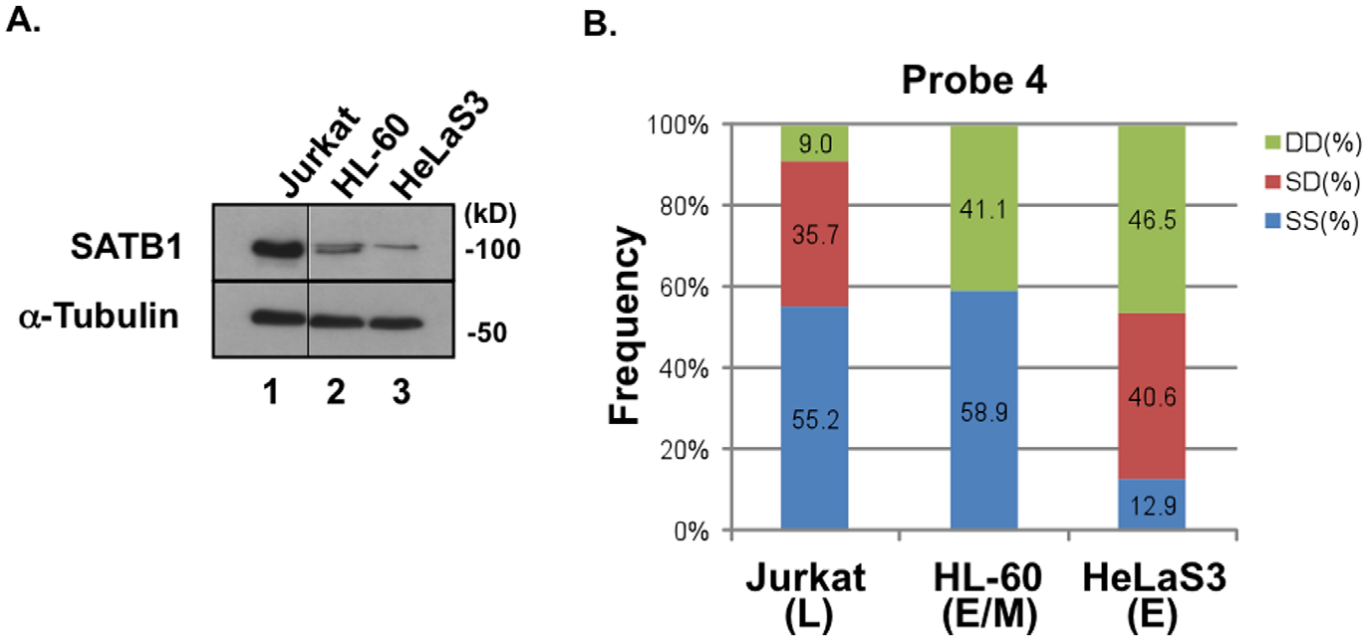
Correlation between SATB1 expression and replication timing at TTR. **A.** SATB1 expression in Jurkat, HL-60 and HeLaS3. Whole cell extracts, separated on 7.5% SDS-polyacrylamide gel, were blotted with anti-SATB1 antibody. Expression of SATB1 is high in T cell (Jurkat), and low or non-detectable in non-T cells (HL-60 and HeLaS3). **B.** Replication timing of the Probe 4 (see **Fig. 2B** and **Table S1**) in Jurkat, HL-60 and HeLaS3. This locus replicates in late-S in Jurkat (high SATB1) and in early-S in HL-60 (low SATB1) and HeLaS3 (very low SATB1).

**Figure 4:**
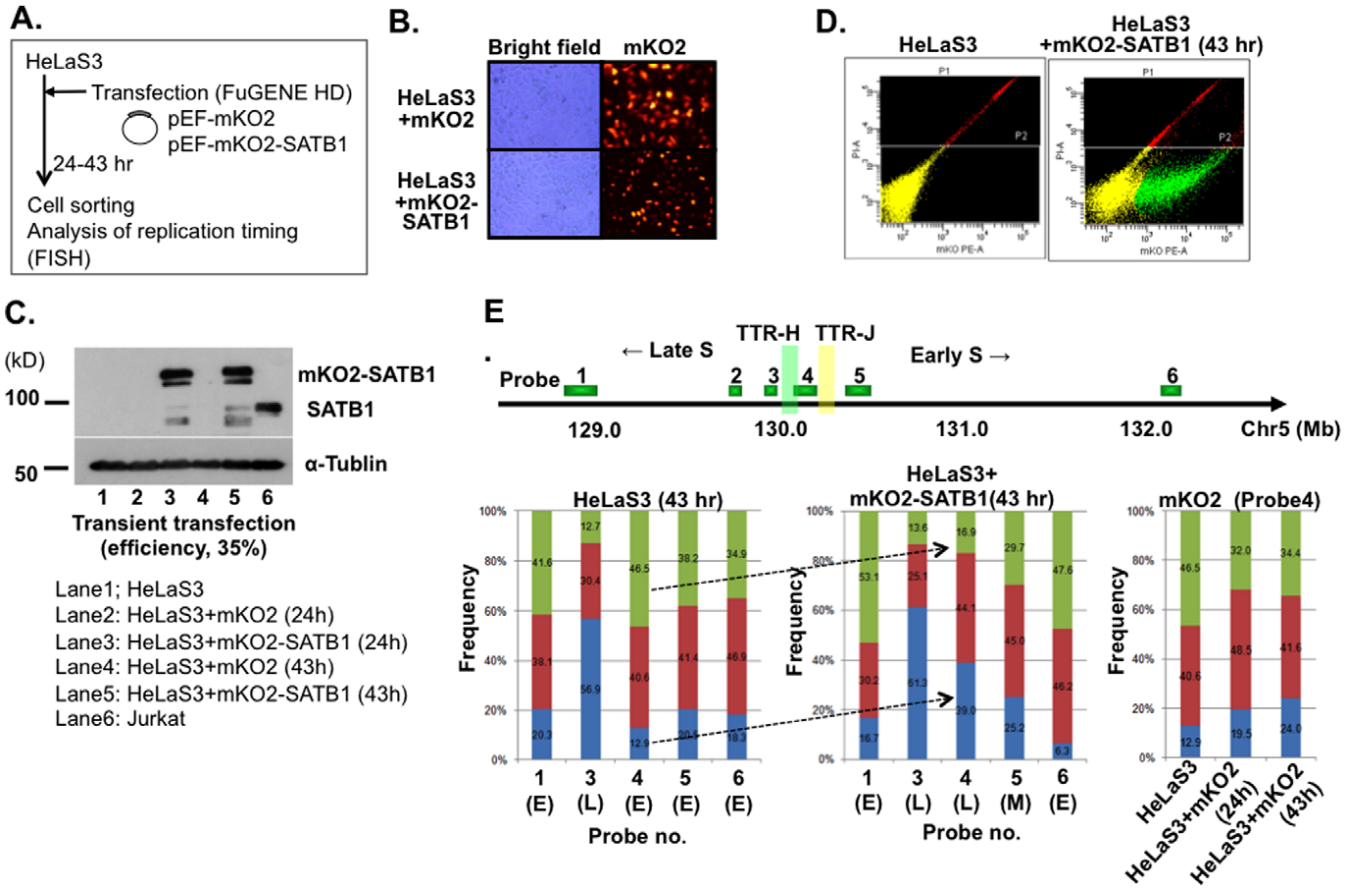
Effect of SATB1 expression on replication timing at TTR: expression of SATB1 in HeLaS3 cells. **A.** The procedure for obtaining HeLaS3 cells overexpressing SATB1. **B.** At 24 hr after transfection with pEF-mKO2 (upper panels) or pEF-mKO2-SATB1 (lower panels), cells were observed using a fluorescence microscope BioZero (KEYENCE). mKO2 fluorescence was observed mainly in the cytoplasm of HeLaS3 cells expressing mKO2 vector or in the nuclei of those expressing mKO2-SATB1. Red, mKO2 signal. **C.** Whole cell extracts were examined by western blotting using anti-SATB1 antibody. Lane 1, non-transfected HeLaS3, lanes 2 and 4, HeLaS3 transfected with pEF-mKO2; lanes 3 and 5, HeLaS3 transfected with pEF-mKO2-SATB1, lane 6, Jurkat. Lanes 2 and 3, 24 hr after transfection; lanes 4 and 5, 43 hr after transfection. **D.** Sorting of mKO2 positive cells by FACS Aria (BD Biosciences). Cells expressing mKO2-SATB1 were separated into viable (yellow) and dead (red) cells stained with PI, then mKO2 positive cells (green) among viable cells were further separated. **E.** Replication timing of HeLaS3 and HeLaS3 expressing mKO2-SATB1 at 43 hr after transfection. We analyzed replication timing of HeLaS3 transfected with mKO2-SATB1 at 24 and 43 hr after transfection. Only the data at 43 hr are shown for cells non-transfected (left panel) or transfected with mKO2-SATB1 plasmid (central panel). At least 200 BrdU-positive nuclei (S-phase) were counted for each probe. Replication timing in TTR (detected by Probe 4) changed from early (HeLaS3) to late (HeLaS3 expressing mKO2-SATB1) (indicated by the arrows). Expression of mKO2 did not affect the replication timing of the transition region (right panel).

To explore whether replication timing can be altered by SATB1, we examined SATB1 expression and replication timing of the TTR in Jurkat, HL-60 and HeLaS3. SATB1 was expressed in a cell type-specific manner, high in Jurkat, low in HL-60, and almost none in HeLaS3 (**Fig. 3A**). FISH analysis was performed by using the probe 4, because this probe permitted the detection of cell-type specific TTRs. We found that replication timing at this locus is late in Jurkat: the frequencies of SS/DD are 55.2/9.0%. On the other hand, in HeLaS3 or HL60, DD or D is 46.5% or 41.1%, respectively, indicating that this region is early-replicating or early/mid-replicating (**Fig. 3B** **and** **Fig. 4E**). Thus, regulation at TTR is correlated with the expression level of SATB1, suggesting a possibility that SATB1 may play a role in determining the timing transition.

### Replication Timing in the Vicinity of the TTR is Affected by SATB1

To evaluate the possibility that SATB1 regulates TTR, we examined the effect of modulation of the SATB1 level on the location of TTR. We first expressed SATB1 in HeLa cells, whose endogenous expression level is very low (**Fig. 4A**). The human SATB1 cDNA was cloned into the CSII-EF-mKO2 vector plasmid [32], generating huSATB1 tagged with mKO2 at its N-terminus (**Fig. 4A**). At 24 and 43 hrs after transfection into HeLaS3 cells, we observed the fluorescent signals of mKO2-SATB1 in nuclei (**Fig. 4B**), whereas those from vector-transfected cells were detected in the cytoplasm. Such patterns of localization were identical to a previous study [42]. Expression of the mKO2-SATB1 protein was confirmed also by western blot analysis (**Fig. 4C**). At 43 hrs after transfection, over 80% of HeLaS3 cells transfected with plasmids were alive and the transfection efficiency was generally around 26∼41%. Although overexpression of SATB1 was reported to be toxic in human cells [43], viability was not significantly affected at least for 48 hrs after transfection under the conditions employed (**Fig. 4D**). Thus, we used HeLaS3 transfected with CSII-EF-mKO2-SATB1 and CSII-EF-mKO2 for further analysis of replication timing.

We collected live and mKO2-positive cells by FACS sorting, which were analyzed by FISH to determine replication timing. In HeLaS3, we observed two types of cells, one containing two homologues and the other containing three homologues at 5q23/31. We first counted signal patterns in over 100 nuclei of two homologue cells (**Fig. 4E**; shown by arrows). In HeLaS3 expressing mKO2-SATB1, replication timing at the probe 4 locus exhibited a dramatic change from early- to late-replicating (DD, 46.5%−>16.9%; SS, 12.9%−>39.0%). We also analyzed the cells with three copies of the locus, and similar change was observed (**Fig. S4**; DDD, 32.5%−>12.7%; SSS, 9.9%−>22.7%). In contrast, replication timing at other probes (probes 3 and 5) did not differ significantly between HeLaS3 and HeLaS3 expressing mKO2-SATB1. At the probes 1 and 6, replication timing became slightly earlier when mKO2-SATB1 was expressed (DD 41.6%−>53.1% and SS 20.3%−>16.7% for probe 1; DD 34.9%−>47.6% and SS 18.3%−>6.3% for probe 6). As a control, we also examined replication timing in HeLaS3 expressing mKO2 (**Fig. 4E**). Although slight change was detected at the probe 4 (DD 46.5%−>34.4%, SS 12.9%−>24.0%), it was much less significant than that observed with mKO2-SATB1. Basically, very similar results were obtained in analyses of three homologue cells (data not shown). These results indicate that expression of SATB1 in HeLa cells results in change of TTR by delaying the replication timing of the Probe 4 locus.

**Figure 5:**
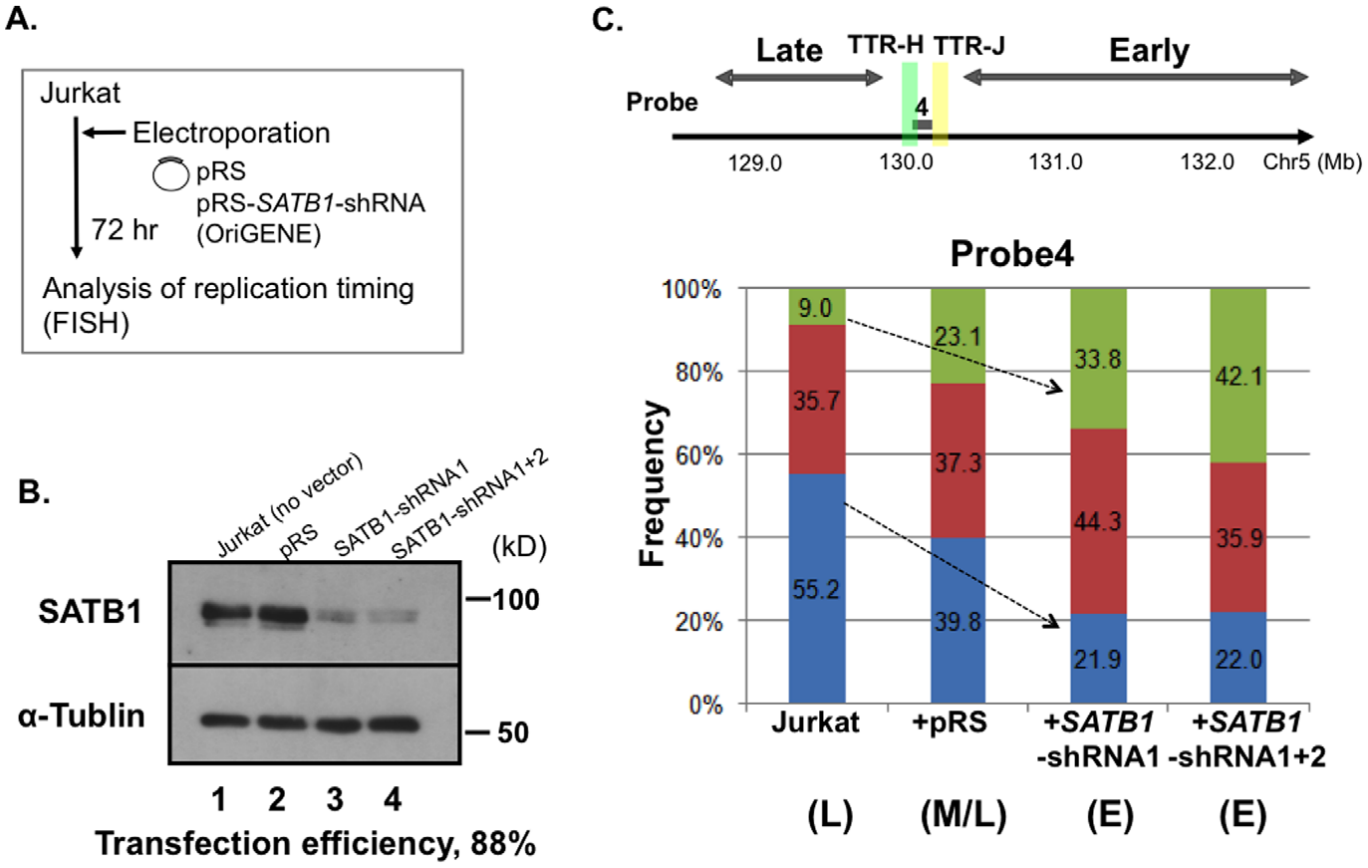
Effect of SATB1 expression on replication timing at TTR: suppression of SATB1 expression in Jurkat cells. **A.** The procedure for repression of SATB1 expression in Jurkat. **B.** Whole cell extracts were examined by western blotting using anti-SATB1 antibody. Lane 1, untransfected Jurkat; lane 2, Jurkat transfected with pRS vector; lane 3 and 4, Jurkat transfected with pRS-SATB1-shRNA1 and with pRS-SATB1-shRNA1+ pRS-SATB1-shRNA2, respectively. Cells were harvested at 72 hr after transfection. **C.** Replication timing was determined by FISH across the TTR. Only the data at the Probe 4 are shown. Replication timing in the transition region changed from late (Jurkat) to early (SATB1-depleted Jurkat) (indicated by the arrows). At least 200 BrdU-positive nuclei (S-phase) were counted.

We next examined the effect of SATB1 knockdown on TTR in Jurkat cells (**Fig. 5A**). shRNA-expressing vectors (pRS-*SATB1*-shRNA1, pRS-*SATB1*-shRNA1+2 [mixture of pRS-*SATB1*-shRNA1 and 2] or pRS vector) were introduced into Jurkat cells by electroporation. Western blotting of the extracts at 72 hr after electroporation confirmed the suppression of SATB1 expression (**Fig. 5B**). Since efficiency of electroporation was over 88%, we used all the cells for FISH without cell sorting (**Fig. 5C**). Replication timing at the Probe 4 locus changed from late to early after repression of SATB1 expression in Jurkat cells (DD 9.0%−>33.8 or 42.1%, SS 55.2%−>21.9% or 22.0%). Electroporation of pRS plasmid only marginally affected the timing at this locus. These results indicate that suppression of SATB1 expression in Jurkat results in change of TTR by causing the Probe 4 locus to become early-replicating. Taken together, above results indicate that SATB1 protein regulates the location of TTR by somehow delaying the replication timing of a specific genome segment.

### SATB1 Binds to the Edge of an Early Replicating Domain Adjacent to TTR

**Figure 6:**
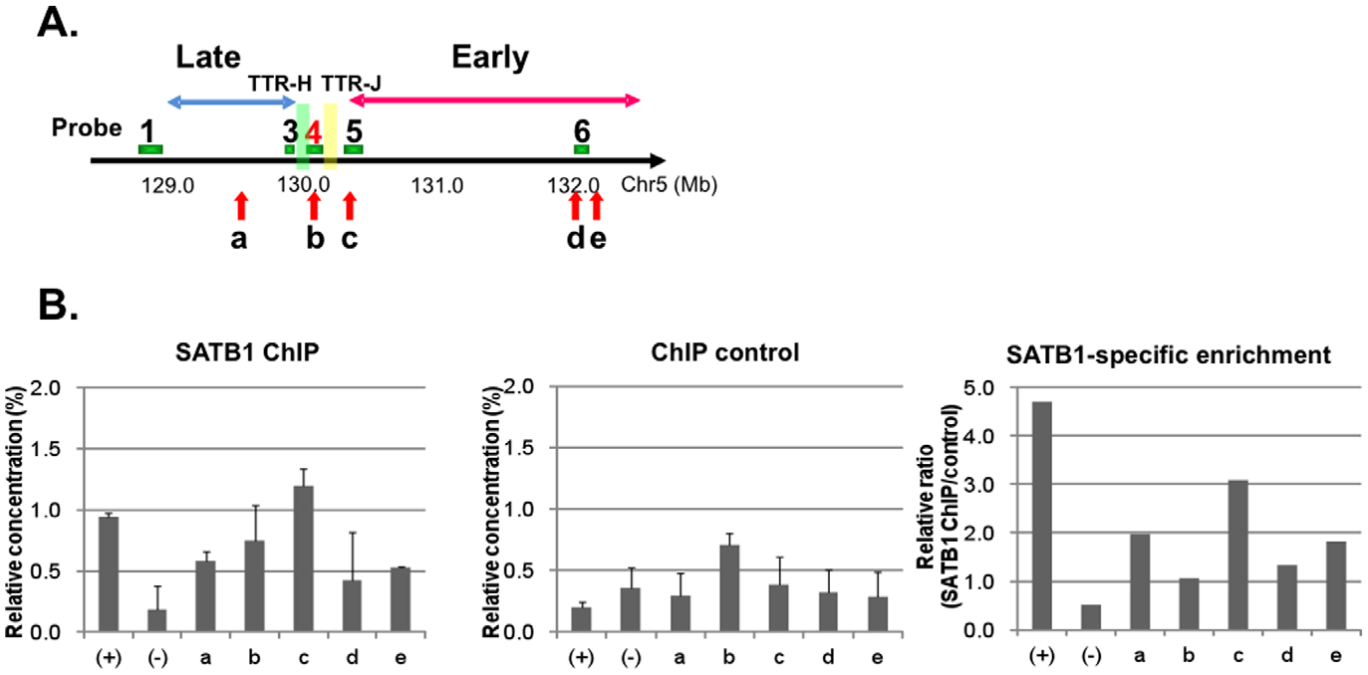
Chromatin immunoprecipitation (ChIP) assays of SATB1 binding. **A.** Locations of primers (a–e) used for ChIP assays. **B.** ChIP analyses were carried out by using anti-mouse SATB1 antibody (left panel) or purified mouse IgG1 control antibody (central panel) jn HeLaS3 cells stably expressing SATB1. Chromain-immunoprecipitated DNA was purified by MinElute (QIAGEN) and used for quantitative PCR. Error bars represent the mean and standard deviations based on four independent experiments. Relative ratio (SATB1/control) is shown as SATB1-specific binding (right panel).

We then examined whether SATB1 binds to TTR or its vicinity as was predicted from the informatics analyses. We employed chromatin immunoprecipitation assays using HeLaS3 cells stably expressing mKO2-SATB1 (**Fig. 6** **)**. Since HeLaS3 cells express SATB1 only at a very low level (**Fig. 3** **and** **4**), SATB1 binding to DNAs in the stable clone occurred almost exclusively by exogenous SATB1. By using the lentivirus transduction system and mKO2-fused expression vectors [32], we could obtain stable clones expressing SATB1 to a significant level without much effect on growth rate and cell cycle (**Fig. S5**), in spite of previous reports that SATB1 overexpression is toxic in human cultured cells [43], and that efficient translation may require the 442 bp SATB1 3′UTR [42]. The level of mKO2-SATB1 was comparable to that of SATB1 in Jurkat cells (data not shown).

SATB1 binds to the 200-kb T-helper 2 (Th2) cytokine locus on the mouse chromosome 11, which is syntenic to the human 5q23/31 examined in this study, and regulates coordinated expression of *Il5*, *Il4* and *Il13* in mice [25]. In human Jurkat T cells, SATB1 binds to the distal promoter region of human *IL-2* and regulates *IL-2* expression, while it does not bind to the proximal promoter region of *IL-2Rα* [41], [44]–[47]. ChIP analyses using anti-SATB1 antibody showed that SATB1 bound to the promoter region of human *IL-2* (positive control) but not to that of *IL-2Rα* (negative control) in Jurkat T cells [46], [47]. We then examined SATB1 binding to the human 5q23/31 region using five different primers; primer a located in late replicating region, primer b located at the cell-type specific TTR, primer c located at the boundary of early-replicating domain, primers d and e located in the early-replicating region near the cytokine genes (**Table S5**). We found that SATB1 specifically bound to the primer c locus, which is located at the edge of the early-to-late transition region in Jurkat cells (**Fig. 6B**). Furthermore, the primer c locus includes the sequences highly similar to the SATB1 consensus (V$SATB1.01; wntAATAnwnnwnn, from Genomatix, not within LINE1) conserved among mammals (**Fig. S3**). These results suggest that SATB1 regulates TTR by directly binding to the early-replicating-domain-proximal boundary.

### SATB1 May Delay the Replication Timing at TRR by Slowing Down the Fork Movement

Above results suggest that SATB1 delays the replication timing in the TTR segment by directly binding to the edge of the early-replicating domain adjacent to TTR. We therefore examined rate of replication by directly measuring the copy number of genome DNA in a synchronous culture. Cells (HeLaS3 and HeLaS3 stably expressing SATB1; **Fig. S5A**) were synchronized at the G1/S boundary by double thymidine block and then the same numbers (0.5×10^6^ cells) of cells were released into cell cycle in a synchronous manner. Total genomic DNA was prepared at different timepoints after release. The amount of the bulk DNA, as measured by FACS analysis, increased with a similar time course after release and reached 4C at 8 hrs in both cells (data not shown), indicating that the proliferation and overall S phase progression are not affected by expression of SATB1 (**Fig. S5B and C**).

**Figure 7:**
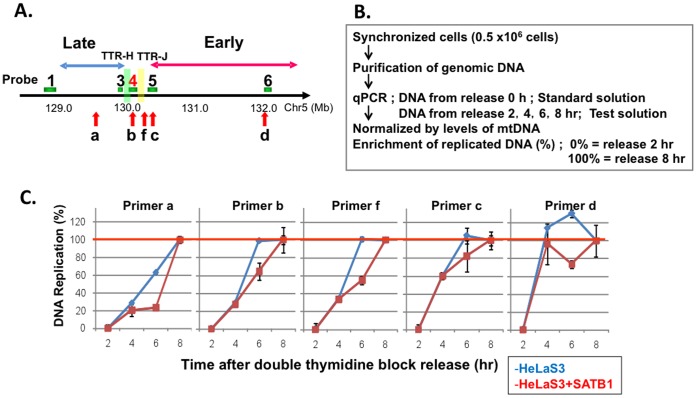
Genome copy number analyses of TRR in synchronized cell population. **A.** Locations of primers (a, b, c, d and f) used for copy number analyses. **B.** The procedure for quantification of copy number. **C**. Time course of DNA replication at various locations in and around TTR. Genome copy numbers of cells synchronously growing from double thymidine block were quantified at various loci at various timepoints. The level of DNA synthesis at 2 hr or 8 hr after release was set as 0% or 100% DNA synthesis, respectively.

We selected 5 loci in and around TTR and changes of copy number were monitored at 0 to 8 hrs after release. Genomic DNA purified at the time of release (0 hr) was used as a standard for quantification (unreplicated DNA). Then, genomic DNAs purified from 2–8 hrs after release were quantified by qPCR at the 5 loci. The amount of genomic DNA from 2–8 hrs after release at each loci was normalized by that at mitochondria region. To estimate the amount of DNA synthesis at each timepoint, the levels of DNA synthesis at 2 and 8 hrs were set at 0% and 100%, respectively (**Fig. 7**).

Replication timing as well as the rate of fork movement could be estimated from this analysis. At the primer d locus, 100% DNA synthesis is achieved at 4 hrs after release in both cells, indicating that this segment is very early-replicating and its replication timing is not affected by SATB1. In contrast, at the primer c, f and b loci, the timing is still early-replicating in HeLaS3, but the 8 hrs is needed to achieve 100% DNA synthesis in SATB1-expressing cells. DNA synthesis appears to proceed slower in the latter cells compared to the former cells. At the primer a locus, 8 hrs is needed to achieve 100% DNA synthesis in HeLaS3, consistent with this loci being late-replicating. In SATB1-expressing HeLaS3, little DNA synthesis is observed at the primer a locus by 6 hrs, and is completed by 8 hrs, indicating that replication timing is further delayed.

Possible mechanisms for delayed replication in TTR and the surrounding region in the presence of SATB1 include the suppression of origin firing in the TTR segment [48] and/or inhibition of fork movement along the TTR. Since it has been proposed that TTR may be composed of a long origin-less segment replicated by a single unidirectional fork [40], and previous mapping did not show the presence of origins in this TTR segment [16], [49]–[52], we speculate that it is most likely that SATB1 slows down the replication fork movement.

## Discussion

Eukaryotic genomes are replicated under a program that dictates the preferred locations for initiation and timing of replication during S phase [2]. In yeasts, checkpoint functions regulate the origin firing program, suppressing the firing of some origins, which may be late-firing or dormant under normal conditions [4], [53]–[56]. Histone modification and transcription factors have also been shown to regulate the origin firing program [10], [11]. In yeast, physiological conditions can also affect the origin selection or timing regulation, suggesting significant plasticity of this process [57]. Single cell or single molecule analyses indicate that origin selection can vary in individual cells and during successive cell cycle in a single cell, suggestive of stochastic nature in the process [58], [59].

In higher eukaryotes, timing of origin firing may be determined by the chromosome domains of Mb size, known as “replication (timing) domain” [12], [13]. The replication domain structures correlate with transcriptional activity, histone modification, and chromatin proximity defined by Hi-C analyses (chromosome conformation capture), and may undergo major change during the differentiation process [12], [15]. As much as 50% of the entire human genome may exhibit cell-type specific replication timing domain structures [16]. However, mechanisms regulating replication timing in mammals have been largely elusive.

The human 5q23/31 locus contains clusters of cytokine genes and presents an attractive model for the studies on regulation of replication timing because of the availability of ample information on transcriptional regulation and chromatin regulation. We previously reported identification of two origins (GM-CSF *ori1* and *ori2*) on human 5q31.1 [21] as well as another new origin downstream of IL-13 (*ori*_IL-13_) and crucial role of CNS (conserved non-coding sequence essential for transcriptional regulation of cytokine genes) [60] for firing of this origin, suggesting possible coregulation of transcriptional activation and origin firing [20], [22]. We also showed that the replication timing of the cytokine locus is not affected by inactivation of this origin, showing that local origin activity does not affect the timing regulation. In this communication, we have compared the replication timing of the 3.5 Mb segment containing the cytokine clusters in T-cells and non-T cells. We have identified difference in timing at the boundary between the early-replicating and late-replicating segments. Genomic features of TTR and its surrounding region suggest the followings. 1) TTR is present in an AT-rich area, adjacent to GC-rich early-replicating segments. 2) TTR coincides with the boundary of synteny. 3) TTR resides in a gene-poor, origin-less region.

We noted the presence of clusters of potential binding sites for SATB1, a T-cell specific transcription regulator, in and near TTR. SATB1 was originally discovered as a T cell-specific AT-rich sequence binding protein, and was shown to bind to MAR [23], [40], [61], [62]. It was shown to regulate T cell activation by recruiting HDAC or HAT to chromatin [45], [46], [63], [64]. It may promote chromatin loop structures for coregulation of cytokine-related genes [24], [25], [65], [66]. It was also shown to promote breast tumor growth and metastasis through regulation of a large number of genes predominantly involved in cell adhesion, cellular signaling and cell cycle [67]. It also contributes to initiation of X inactivation [68]–[70], and may regulate embryonic stem cell differentiation through modulation of Nanog expression [71]. We found that the level of SATB1 correlates with the location of the replication timing boundary. Therefore, we decided to examine potential roles of SATB1 in determination of replication timing domains. We conducted two experiments. The first was to examine the effect of SATB1 expression in HeLaS3 cells which normally do not express SATB1. The second was to repress the expression of SATB1 in Jurkat cells which normally express SATB1 at a high level. We have shown in both experiments that SATB1 expression correlates with the extension of the late-replicating segment toward the early-replicating segment, indicating that SATB1 somehow enforces the late replication at TTR. By ChIP assays, we showed that SATB1 indeed binds to the vicinity of the TTR.

We then measured rate of DNA synthesis through S phase at or near TTR by measuring the copy number increase and showed that the rate of DNA synthesis is delayed at the TTR where SATB1 binds. This could be due to failure of firing at the origins potentially present at TTR or its surrounding region. However, it has been known that replication timing boundary is generally associated with origin-less segment, and previous analyses have indicated the absence of origins in this TTR [48], [49]. Therefore, we speculate that fork progression may be slowed down or arrested by SATB1. We do not know how SATB1 interferes the progression of a replication fork at the moment. SATB1 may bind to some nuclear structures (nuclear matrix or skelton), as was previously indicated [23], [40], [61], [62], and this may interfere with fork progression. We have detected change of histone modification near TTR in the presence or absence of SATB1. Generally, active marks increase in the absence of SATB1, whereas they decrease in the presence of SATB1 (from UCSC genome browser), and this may be correlating with early or late replication, respectively. However, SATB1 is also involved in activation of transcription through inducing histone acetylation [24], [45], [63], [64].

What would be the physiological significance of regulation of replication timing by SATB1? SATB1 does not seem to affect genome-wide replication domain structures (data not shown). Rather, it affects local replication timing where it binds. Slowed replication in the presence of SATB1 may be a result of chromatin modification or altered transcriptional activity. Alternatively, SATB1-mediated arrest/pausing of replication fork may contribute to more error-free replication of TTR, which tends to be associated with higher mutation rate and more SNP. As suggested before, SATB1 could facilitate the formation of chromatin loop structures [25], [66], [72]–[75], which could be intimately related to transcriptional regulation as well as to replication timing domain structures. Although SATB1 does not appear to play a major role in defining the general replication timing domain structures (our unpublished data), it may fine-tune the replication timing so that replication and transcription may be coordinately regulated. Genome-wide views on how SATB1 affects replication timing as well as on the binding sites of SATB1 would help elucidate the precise roles of SATB1 in regulation of DNA replication.

## Supporting Information

Figure S1 **Replication timing of the human 5q23/31.** Replication timing assays were conducted as described in the legend to Fig. 1. The locations of the primers used are indicated along the 5q23/31 region shown to the right of the panels. Probes 1–6 represent those used in FISH assays (**Fig. 2**), and red boxes show the peak timing fraction. See **Table S1** for the locations of the primers.
(TIF)

Figure S2 **Genomic features of the human 5q23/31 region.** **A.** Chromosomal synteny among human, rat, mouse and chicken corresponding to the human 5q23/31 region. **B.** Distribution of GC content (%). The GC contents were calculated by sliding window analyses across the genome region shown (sliding size, 1 kb; window size, 10–100 kb). The result indicates that early replicating segment is located in higher GC region (>43%). On the other hand, late replicating region as well as transition region are located in low GC region (<43%). **C.** Distribution of LINEs. A segment containing the most LINE1 was identified near the TTR (32 LINEs in the 25 kb segment, 130.025–130.05 Mb; red arrow). **D.** Distribution of SINEs. Average numbers of SINEs were calculated within the early- and late-replicating domains and are shown as horizontal red bars (19 SINEs/25 kb and 8 SINEs/25 kb, in early and late regions, respectively). Data were extracted from a RepeatMasker analysis (http://www.repeatmasker.org/) on the 129.0–132.5 Mb segment of the human chromosome 5. The numbers of LINE1 and SINEs were calculated by a sliding window analysis (sliding size: 25 kb, window size: 25 kb). **E.** Gene density. The total numbers of genes were calculated in non-overlapping windows of 250 kb each. The genes used in the analysis were taken from NCBI *H. sapiens* Genome (http://www.ncbi.nlm.nih.gov/). The gene density in the early replicating region is significantly higher than that in the late replicating region. In the vicinity of the IL-13/IL-4 gene loci, there are 11 genes in a 250 kb segment (130–132.25). Notably, no experimentally confirmed genes are present in the TTR. TTR-H and TTR-J are shown by green and yellow vertical bars, respectively.
(TIF)

Figure S3 **Distribution of potential SATB1 binding sequences (SBSs) at the human 5q23/31.** **A.** Sequences similar to SBS-1∼16 on the 129–132.5Mb segment of human chromosome 5 were searched by using NCBI Blast (http://www.ncbi.nlm.nih.gov/BLAST/). Sequences similar to SBS-2 (514 bp), −4 (465 bp) and −14 (387 bp) were found, and “bit scores” were plotted against identity. **B.** Sequences with high identity (≧85%) and bit scores (≧400) as “highly similar” SBSs were selected (0 sequence for SBS-2, 9 sequences for SBS-4 and 39 sequences of SBS-14). Locations and numbers of these potential SBSs are shown on the human 5q23/31 3.5-Mb segment. **C.** Among them, nine pairs of SBS-4 and -14 that were found to be present on one Line1P (highlighted in yellow in Table S4) are indicated (blue, green and yellow small rectangles). Five of them were identified to be present close to the TTR (shown within the orange box). These findings lead us to suggest a possibility that the SBS clusters within and close to TTR may play a role in defining the replication timing boundary. Different colors for the rectangles indicate different orientations of SBS-4 and -14 on each Line1P. TTR-H and TTR-J are shown by green and yellow vertical bars, respectively.
(TIF)

Figure S4 **Effect of SATB1 expression on replication timing at TTR in HeLaS3 cells (with three chromosomes at the 5q locus).** Replication timing of HeLaS3 and HeLaS3 expressing mKO2-SATB1 at 43 hr after transfection. We analyzed replication timing of HeLaS3 transfected with mKO2-SATB1 at 24 and 43 hr after transfection. Only the data at 43 hr are shown for cells non-transfected (left panel) or transfected with mKO2-SATB1 plasmid (right panel). At least 200 BrdU-positive nuclei (S-phase) containing three chromosomes at the 5q locus were counted for Probe 3 and 4. Replication timing in TTR (detected by Probe 4) changed from early (HeLaS3) to mid/late (HeLaS3 expressing mKO2-SATB1) (indicated by the arrows).
(TIF)

Figure S5 **Cell growth and cell cycle of HeLaS3 and HeLaS3 cells stably expressing mKO2-SATB1.** **A.** HeLaS3 and HeLaS3 cells stably expressing mKO2-SATB1 were observed by FSX100 (OLYMPUS). Red, mKO2 signal. **B.** Growth rate of HeLaS3 and stable mKO2-SATB1 HeLaS3. **C.** Stable mKO2-SATB1 HeLaS3 cells were synchronized at the G1/S boundary by double thymidine block, and then synchronously released into cell cycle and were collected at the times indicated (0, 2, 4, 6, 8 and 9 hrs). Collected cells were fixed in 70% ethanol, and DNA contents were analyzed by FACS. Growth rate and cell cycle distribution are not affected by expression of SATB1 in HeLaS3 cells.
(TIF)

Table S1 Locations of PCR primers used to determine replication timing at the human 5q23/31. Primers located further to the right of position (9) are not listed in this table.
(TIF)

Table S2 BAC clones used for replication timing analyses by FISH.
(TIF)

Table S3 The names of the genes at the human 5q23/31. and their map positions and gene sizes.
(TIF)

Table S4 Locations of sequences highly similar to known SATB1 binding sites located on L1P of the LINE-1 subfamily. Yellow columns indicate the Line1P sequences that carry both SBS-4 and 14, which are shown in Fig. S3C.
(TIF)

Table S5 Locations of the primer sets used for ChIP analyses of SATB1 binding and for copy number analyses.
(TIF)

## References

[1] Arias EE, Walter JC (2007) Strength in numbers: preventing rereplication via multiple mechanisms in eukaryotic cells. Genes Dev 21: 497–518. 17344412 10.1101/gad.1508907

[2] Masai H, Matsumoto S, You Z, Yoshizawa-Sugata N, Oda M (2010) Eukaryotic chromosome DNA replication: where, when, and how? Annu Rev Biochem 79: 89–130. 20373915 10.1146/annurev.biochem.052308.103205

[3] Gilbert DM (2010) Evaluating genome-scale approaches to eukaryotic DNA replication. Nat Rev Genet 11: 673–684. 20811343 10.1038/nrg2830 PMC2962615

[4] Hayano M, Kanoh Y, Matsumoto S, Renard-Guillet C, Shirahige K, et al (2012) Rif1 is a global regulator of timing of replication origin firing in fission yeast. Genes Dev 26: 137–150. 22279046 10.1101/gad.178491.111 PMC3273838

[5] Merrick CJ, Jackson D, Diffley JF (2004) Visualization of altered replication dynamics after DNA damage in human cells. J Biol Chem 279: 20067–20075. 14982920 10.1074/jbc.M400022200

[6] Seiler JA, Conti C, Syed A, Aladjem MI, Pommier Y (2007) The intra-S-phase checkpoint affects both DNA replication initiation and elongation: single-cell and -DNA fiber analyses. Mol Cell Biol 27: 5806–5818. 17515603 10.1128/MCB.02278-06 PMC1952133

[7] Chastain PD, Heffernan TP, Nevis KR, Lin L, Kaufmann WK, et al (2006) Checkpoint regulation of replication dynamics in UV-irradiated human cells. Cell Cycle 5: 2160–2167. 16969085 10.4161/cc.5.18.3236

[8] Karnani N, Dutta A (2011) The effect of the intra-S-phase checkpoint on origins of replication in human cells. Genes Dev 25: 621–633. 21406556 10.1101/gad.2029711 PMC3059835

[9] Wu PY, Nurse P (2009) Establishing the program of origin firing during S phase in fission Yeast. Cell 136: 852–864. 19269364 10.1016/j.cell.2009.01.017 PMC2787407

[10] Aparicio JG, Viggiani CJ, Gibson DG, Aparicio OM (2004) The Rpd3-Sin3 histone deacetylase regulates replication timing and enables intra-S origin control in Saccharomyces cerevisiae. Mol Cell Biol 24: 4769–4780. 15143171 10.1128/MCB.24.11.4769-4780.2004 PMC416400

[11] Knott SR, Peace JM, Ostrow AZ, Gan Y, Rex AE, et al (2012) Forkhead transcription factors establish origin timing and long-range clustering in S. cerevisiae. Cell 148: 99–111. 22265405 10.1016/j.cell.2011.12.012 PMC3266545

[12] Hiratani I, Ryba T, Itoh M, Yokochi T, Schwaiger M, et al (2008) Global reorganization of replication domains during embryonic stem cell differentiation. PLoS Biol 6: e245. 18842067 10.1371/journal.pbio.0060245 PMC2561079

[13] Ryba T, Hiratani I, Lu J, Itoh M, Kulik M, et al (2010) Evolutionarily conserved replication timing profiles predict long-range chromatin interactions and distinguish closely related cell types. Genome Res 20: 761–770. 20430782 10.1101/gr.099655.109 PMC2877573

[14] Birney E, Stamatoyannopoulos JA, Dutta A, Guigó R, Gingeras TR, et al (2007) Identification and analysis of functional elements in 1% of the human genome by the ENCODE pilot project. Nature 447: 799–816. 17571346 10.1038/nature05874 PMC2212820

[15] Hiratani I, Ryba T, Itoh M, Rathjen J, Kulik M, et al (2010) Genome-wide dynamics of replication timing revealed by in vitro models of mouse embryogenesis. Genome Res 20: 155–169. 19952138 10.1101/gr.099796.109 PMC2813472

[16] Hansen RS, Thomas S, Sandstrom R, Canfield TK, Thurman RE, et al (2010) Sequencing newly replicated DNA reveals widespread plasticity in human replication timing. Proc Natl Acad Sci U S A 107: 139–144. 19966280 10.1073/pnas.0912402107 PMC2806781

[17] Schwaiger M, Stadler MB, Bell O, Kohler H, Oakeley EJ, et al (2009) Chromatin state marks cell-type- and gender-specific replication of the Drosophila genome. Genes Dev 23: 589–601. 19270159 10.1101/gad.511809 PMC2658520

[18] Lieberman-Aiden E, van Berkum NL, Williams L, Imakaev M, Ragoczy T, et al (2009) Comprehensive mapping of long-range interactions reveals folding principles of the human genome. Science 326: 289–293. 19815776 10.1126/science.1181369 PMC2858594

[19] Göndör A, Ohlsson R (2009) Chromosome crosstalk in three dimensions. Nature 461: 212–217. 19741702 10.1038/nature08453

[20] Hayashida T, Oda M, Ohsawa K, Yamaguchi A, Hosozawa T, et al (2006) Replication initiation from a novel origin identified in the Th2 cytokine cluster locus requires a distant conserved noncoding sequence. J Immunol 176: 5446–5454. 16622012 10.4049/jimmunol.176.9.5446

[21] Todorovic V, Giadrossi S, Pelizon C, Mendoza-Maldonado R, Masai H, et al (2005) Human origins of DNA replication selected from a library of nascent DNA. Mol Cell 19: 567–575. 16109380 10.1016/j.molcel.2005.07.005

[22] Aladjem MI (2007) Replication in context: dynamic regulation of DNA replication patterns in metazoans. Nat Rev Genet 8: 588–600. 17621316 10.1038/nrg2143

[23] Dickinson LA, Joh T, Kohwi Y, Kohwi-Shigematsu T (1992) A tissue-specific MAR/SAR DNA-binding protein with unusual binding site recognition. Cell 70: 631–645. 1505028 10.1016/0092-8674(92)90432-c

[24] Cai S, Han HJ, Kohwi-Shigematsu T (2003) Tissue-specific nuclear architecture and gene expression regulated by SATB1. Nat Genet 34: 42–51. 12692553 10.1038/ng1146

[25] Cai S, Lee CC, Kohwi-Shigematsu T (2006) SATB1 packages densely looped, transcriptionally active chromatin for coordinated expression of cytokine genes. Nat Genet 38: 1278–1288. 17057718 10.1038/ng1913

[26] Beyer M, Thabet Y, Müller RU, Sadlon T, Classen S, et al (2011) Repression of the genome organizer SATB1 in regulatory T cells is required for suppressive function and inhibition of effector differentiation. Nat Immunol 12: 898–907. 21841785 10.1038/ni.2084 PMC3669688

[27] Watanabe Y, Fujiyama A, Ichiba Y, Hattori M, Yada T, et al (2002) Chromosome-wide assessment of replication timing for human chromosomes 11q and 21q: disease-related genes in timing-switch regions. Hum Mol Genet 11: 13–21. 11772995 10.1093/hmg/11.1.13

[28] Hansen RS, Canfield TK, Lamb MM, Gartler SM, Laird CD (1993) Association of fragile X syndrome with delayed replication of the FMR1 gene. Cell 73: 1403–1409. 8324827 10.1016/0092-8674(93)90365-w

[29] Gilbert DM, Cohen SN (1987) Bovine papilloma virus plasmids replicate randomly in mouse fibroblasts throughout S phase of the cell cycle. Cell 50: 59–68. 3036365 10.1016/0092-8674(87)90662-3

[30] Strehl S, LaSalle JM, Lalande M (1997) High-resolution analysis of DNA replication domain organization across an R/G-band boundary. Mol Cell Biol 17: 6157–6166. 9315676 10.1128/mcb.17.10.6157 PMC232466

[31] Nogami M, Nogami O, Kagotani K, Okumura M, Taguchi H, et al (2000) Intranuclear arrangement of human chromosome 12 correlates to large-scale replication domains. Chromosoma 108: 514–522. 10794573 10.1007/s004120050403

[32] Sakaue-Sawano A, Kurokawa H, Morimura T, Hanyu A, Hama H, et al (2008) Visualizing spatiotemporal dynamics of multicellular cell-cycle progression. Cell 132: 487–498. 18267078 10.1016/j.cell.2007.12.033

[33] Yoshizawa-Sugata N, Masai H (2007) Human Tim/Timeless-interacting protein, Tipin, is required for efficient progression of S phase and DNA replication checkpoint. J Biol Chem 282: 2729–2740. 17102137 10.1074/jbc.M605596200

[34] Ansel KM, Djuretic I, Tanasa B, Rao A (2006) Regulation of Th2 differentiation and Il4 locus accessibility. Annu Rev Immunol 24: 607–656. 16551261 10.1146/annurev.immunol.23.021704.115821

[35] Forbes E, van Panhuys N, Min B, Le Gros G (2010) Differential requirements for IL-4/STAT6 signalling in CD4 T-cell fate determination and Th2-immune effector responses. Immunol Cell Biol 88: 240–243. 20010912 10.1038/icb.2009.101

[36] Paul WE (2010) What determines Th2 differentiation, in vitro and in vivo? Immunol Cell Biol 88: 236–239. 20157328 10.1038/icb.2010.2

[37] Arai KI, Lee F, Miyajima A, Miyatake S, Arai N, et al (1990) Cytokines: coordinators of immune and inflammatory responses. Annu Rev Biochem 59: 783–836. 1695833 10.1146/annurev.bi.59.070190.004031

[38] Watanabe Y, Tenzen T, Nagasaka Y, Inoko H, Ikemura T (2000) Replication timing of the human X-inactivation center (XIC) region: correlation with chromosome bands. Gene 252: 163–172. 10903448 10.1016/s0378-1119(00)00208-0

[39] Woodfine K, Fiegler H, Beare DM, Collins JE, McCann OT, et al (2004) Replication timing of the human genome. Hum Mol Genet 13: 191–202. 14645202 10.1093/hmg/ddh016

[40] de Belle I, Cai S, Kohwi-Shigematsu T (1998) The genomic sequences bound to special AT-rich sequence-binding protein 1 (SATB1) in vivo in Jurkat T cells are tightly associated with the nuclear matrix at the bases of the chromatin loops. J Cell Biol 141: 335–348. 9548713 10.1083/jcb.141.2.335 PMC2148460

[41] Kumar PP, Mehta S, Purbey PK, Notani D, Jayani RS, et al (2007) SATB1-binding sequences and Alu-like motifs define a unique chromatin context in the vicinity of human immunodeficiency virus type 1 integration sites. J Virol 81: 5617–5627. 17376900 10.1128/JVI.01405-06 PMC1900249

[42] Nakayama Y, Mian IS, Kohwi-Shigematsu T, Ogawa T (2005) A nuclear targeting determinant for SATB1, a genome organizer in the T cell lineage. Cell Cycle 4: 1099–1106. 15970696

[43] Kohwi-Shigematsu T, Maass K, Bode J (1997) A thymocyte factor SATB1 suppresses transcription of stably integrated matrix-attachment region-linked reporter genes. Biochemistry 36: 12005–12010. 9340009 10.1021/bi971444j

[44] Notani D, Gottimukkala KP, Jayani RS, Limaye AS, Damle MV, et al (2010) Global regulator SATB1 recruits beta-catenin and regulates T(H)2 differentiation in Wnt-dependent manner. PLoS Biol 8: e1000296. 20126258 10.1371/journal.pbio.1000296 PMC2811152

[45] Purbey PK, Singh S, Notani D, Kumar PP, Limaye AS, et al (2009) Acetylation-dependent interaction of SATB1 and CtBP1 mediates transcriptional repression by SATB1. Mol Cell Biol 29: 1321–1337. 19103759 10.1128/MCB.00822-08 PMC2643834

[46] Kumar PP, Purbey PK, Ravi DS, Mitra D, Galande S (2005) Displacement of SATB1-bound histone deacetylase 1 corepressor by the human immunodeficiency virus type 1 transactivator induces expression of interleukin-2 and its receptor in T cells. Mol Cell Biol 25: 1620–1633. 15713622 10.1128/MCB.25.5.1620-1633.2005 PMC549366

[47] Yasui Yasui D, Miyano M, Cai S, Varga-Weisz P, Kohwi-Shigematsu T (2002) SATB1 targets chromatin remodelling to regulate genes over long distances. Nature. 419: 641–645. 10.1038/nature01084 12374985

[48] Guan Z, Hughes CM, Kosiyatrakul S, Norio P, Sen R, et al (2009) Decreased replication origin activity in temporal transition regions. J Cell Biol 187: 623–635. 19951913 10.1083/jcb.200905144 PMC2806585

[49] Farkash-Amar S, Simon I (2010) Genome-wide analysis of the replication program in mammals. Chromosome Res 18: 115–125. 20205353 10.1007/s10577-009-9091-5

[50] Karnani N, Taylor C, Malhotra A, Dutta A (2007) Pan-S replication patterns and chromosomal domains defined by genome-tiling arrays of ENCODE genomic areas. Genome Res 17: 865–876. 17568004 10.1101/gr.5427007 PMC1891345

[51] Karnani N, Taylor CM, Malhotra A, Dutta A (2010) Genomic study of replication initiation in human chromosomes reveals the influence of transcription regulation and chromatin structure on origin selection. Mol Biol Cell 21: 393–404. 19955211 10.1091/mbc.E09-08-0707 PMC2814785

[52] Cadoret JC, Meisch F, Hassan-Zadeh V, Luyten I, Guillet C, et al (2008) Genome-wide studies highlight indirect links between human replication origins and gene regulation. Proc Natl Acad Sci U S A 105: 15837–15842. 18838675 10.1073/pnas.0805208105 PMC2572913

[53] Santocanale C, Diffley JF (1998) A Mec1- and Rad53-dependent checkpoint controls late-firing origins of DNA replication. Nature 395: 615–618. 9783589 10.1038/27001

[54] Shirahige K, Hori Y, Shiraishi K, Yamashita M, Takahashi K, et al (1998) Regulation of DNA-replication origins during cell-cycle progression. Nature 395: 618–621. 9783590 10.1038/27007

[55] Kumar S, Huberman JA (2009) Checkpoint-dependent regulation of origin firing and replication fork movement in response to DNA damage in fission yeast. Mol Cell Biol 29: 602–611. 19001087 10.1128/MCB.01319-08 PMC2612511

[56] Shechter D, Costanzo V, Gautier J (2004) ATR and ATM regulate the timing of DNA replication origin firing. Nat Cell Biol 6: 648–655. 15220931 10.1038/ncb1145

[57] Matsumoto S, Hayano M, Kanoh Y, Masai H (2011) Multiple pathways can bypass the essential role of fission yeast Hsk1 kinase in DNA replication initiation. J Cell Biol 195: 387–401. 22024164 10.1083/jcb.201107025 PMC3206344

[58] Schultz SS, Desbordes SC, Du Z, Kosiyatrakul S, Lipchina I, et al (2010) Single-molecule analysis reveals changes in the DNA replication program for the POU5F1 locus upon human embryonic stem cell differentiation. Mol Cell Biol 30: 4521–4534. 20647538 10.1128/MCB.00380-10 PMC2937526

[59] Courbet S, Gay S, Arnoult N, Wronka G, Anglana M, et al (2008) Replication fork movement sets chromatin loop size and origin choice in mammalian cells. Nature 455: 557–560. 18716622 10.1038/nature07233

[60] Loots GG, Locksley RM, Blankespoor CM, Wang ZE, Miller W, et al (2000) Identification of a coordinate regulator of interleukins 4, 13, and 5 by cross-species sequence comparisons. Science 288: 136–140. 10753117 10.1126/science.288.5463.136

[61] Nakagomi K, Kohwi Y, Dickinson LA, Kohwi-Shigematsu T (1994) A novel DNA-binding motif in the nuclear matrix attachment DNA-binding protein SATB1. Mol Cell Biol 14: 1852–1860. 8114718 10.1128/mcb.14.3.1852-1860.1994 PMC358543

[62] Dickinson LA, Dickinson CD, Kohwi-Shigematsu T (1997) An atypical homeodomain in SATB1 promotes specific recognition of the key structural element in a matrix attachment region. J Biol Chem 272: 11463–11470. 9111059 10.1074/jbc.272.17.11463

[63] Notani D, Limaye AS, Kumar PP, Galande S (2010) Phosphorylation-dependent regulation of SATB1, the higher-order chromatin organizer and global gene regulator. Methods Mol Biol 647: 317–335. 20694677 10.1007/978-1-60761-738-9_20

[64] Pavan Kumar P, Purbey PK, Sinha CK, Notani D, Limaye A, et al (2006) Phosphorylation of SATB1, a global gene regulator, acts as a molecular switch regulating its transcriptional activity in vivo. Mol Cell 22: 231–243. 16630892 10.1016/j.molcel.2006.03.010

[65] Cai S, Kohwi-Shigematsu T (1999) Intranuclear relocalization of matrix binding sites during T cell activation detected by amplified fluorescence in situ hybridization. Methods 19: 394–402. 10579934 10.1006/meth.1999.0875

[66] Yasui D, Miyano M, Cai S, Varga-Weisz P, Kohwi-Shigematsu T (2002) SATB1 targets chromatin remodelling to regulate genes over long distances. Nature 419: 641–645. 12374985 10.1038/nature01084

[67] Han HJ, Russo J, Kohwi Y, Kohwi-Shigematsu T (2008) SATB1 reprogrammes gene expression to promote breast tumour growth and metastasis. Nature 452: 187–193. 18337816 10.1038/nature06781

[68] Agrelo R, Wutz A (2009) Cancer progenitors and epigenetic contexts: an Xisting connection. Epigenetics 4: 568–570. 19923898 10.4161/epi.4.8.10186

[69] Agrelo R, Souabni A, Novatchkova M, Haslinger C, Leeb M, et al (2009) SATB1 defines the developmental context for gene silencing by Xist in lymphoma and embryonic cells. Dev Cell 16: 507–516. 19386260 10.1016/j.devcel.2009.03.006 PMC3997300

[70] Agrelo R, Wutz A (2010) ConteXt of change–X inactivation and disease. EMBO Mol Med 2: 6–15. 20043281 10.1002/emmm.200900053 PMC3377189

[71] Savarese F, Dávila A, Nechanitzky R, De La Rosa-Velazquez I, Pereira CF, et al (2009) Satb1 and Satb2 regulate embryonic stem cell differentiation and Nanog expression. Genes Dev 23: 2625–2638. 19933152 10.1101/gad.1815709 PMC2779756

[72] Gong F, Sun L, Wang Z, Shi J, Li W, et al (2011) The BCL2 gene is regulated by a special AT-rich sequence binding protein 1-mediated long range chromosomal interaction between the promoter and the distal element located within the 3’-UTR. Nucleic Acids Res 39: 4640–4652. 21310710 10.1093/nar/gkr023 PMC3113567

[73] Wang L, Di LJ, Lv X, Zheng W, Xue Z, et al (2009) Inter-MAR association contributes to transcriptionally active looping events in human beta-globin gene cluster. PLoS One 4: e4629. 19247486 10.1371/journal.pone.0004629 PMC2645683

[74] Galande S, Purbey PK, Notani D, Kumar PP (2007) The third dimension of gene regulation: organization of dynamic chromatin loopscape by SATB1. Curr Opin Genet Dev 17: 408–414. 17913490 10.1016/j.gde.2007.08.003

[75] Kumar PP, Bischof O, Purbey PK, Notani D, Urlaub H, et al (2007) Functional interaction between PML and SATB1 regulates chromatin-loop architecture and transcription of the MHC class I locus. Nat Cell Biol 9: 45–56. 17173041 10.1038/ncb1516

