# Supplementary figures and images for: Regulation of DNA Replication Timing on Human Chromosome by a Cell-Type Specific DNA Binding Protein SATB1
Source: PLoS One. 2012 Aug 7;7(8):e42375. doi: 10.1371/journal.pone.0042375 (PMC3413666; doi:10.1371/journal.pone.0042375)

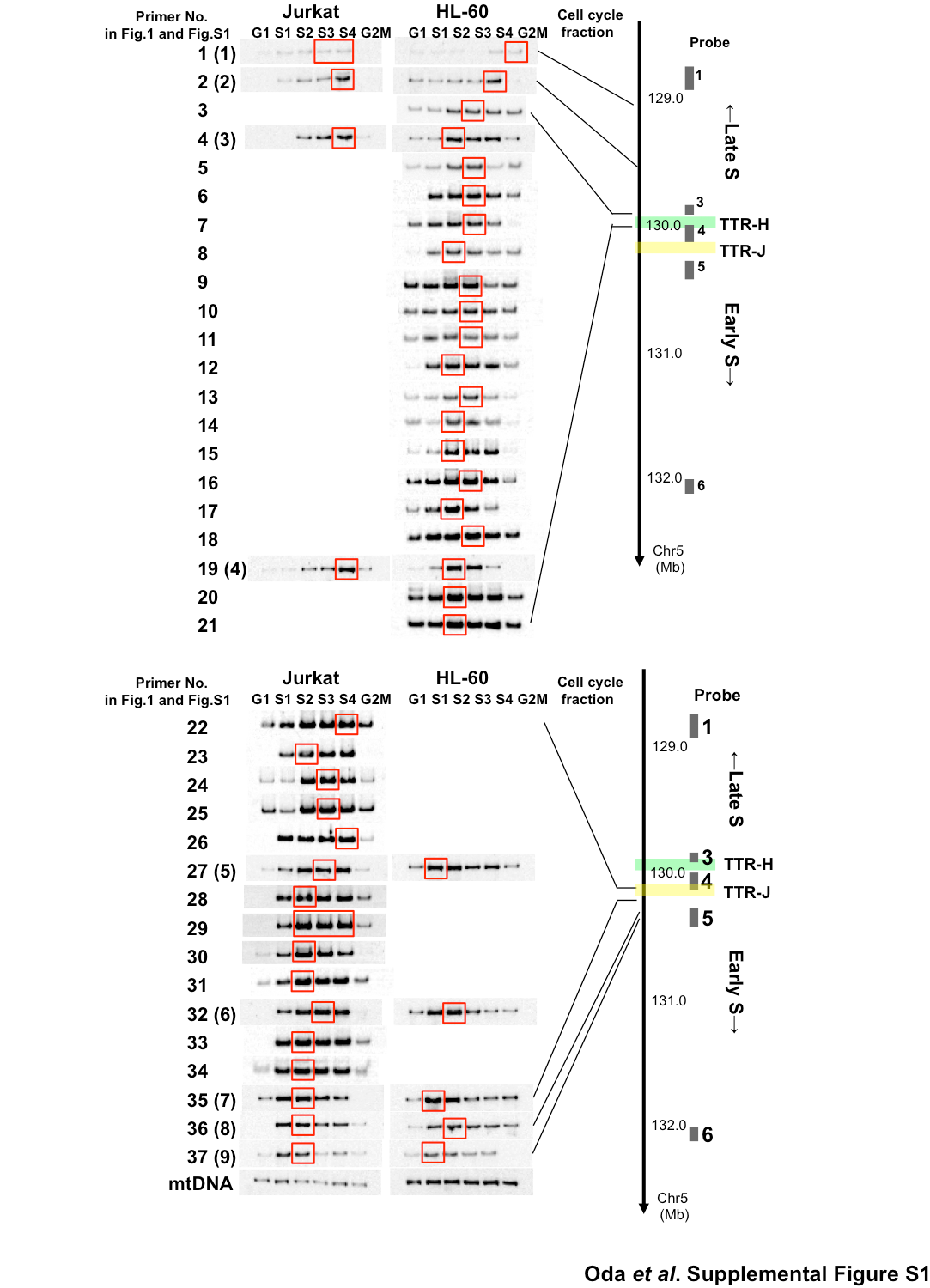

Supplement: Figure S1 — Replication timing of the human 5q23/31. Replication timing assays were conducted as described in the legend to Fig. 1. The locations of the primers used are indicated along the 5q23/31 region shown to the right of the panels. Probes 1–6 represent those used in FISH assays ( Fig. 2 ), and red boxes show the peak timing fraction. See Table S1 for the locations of the primers. (TIF) [file pone.0042375.s001.tif]

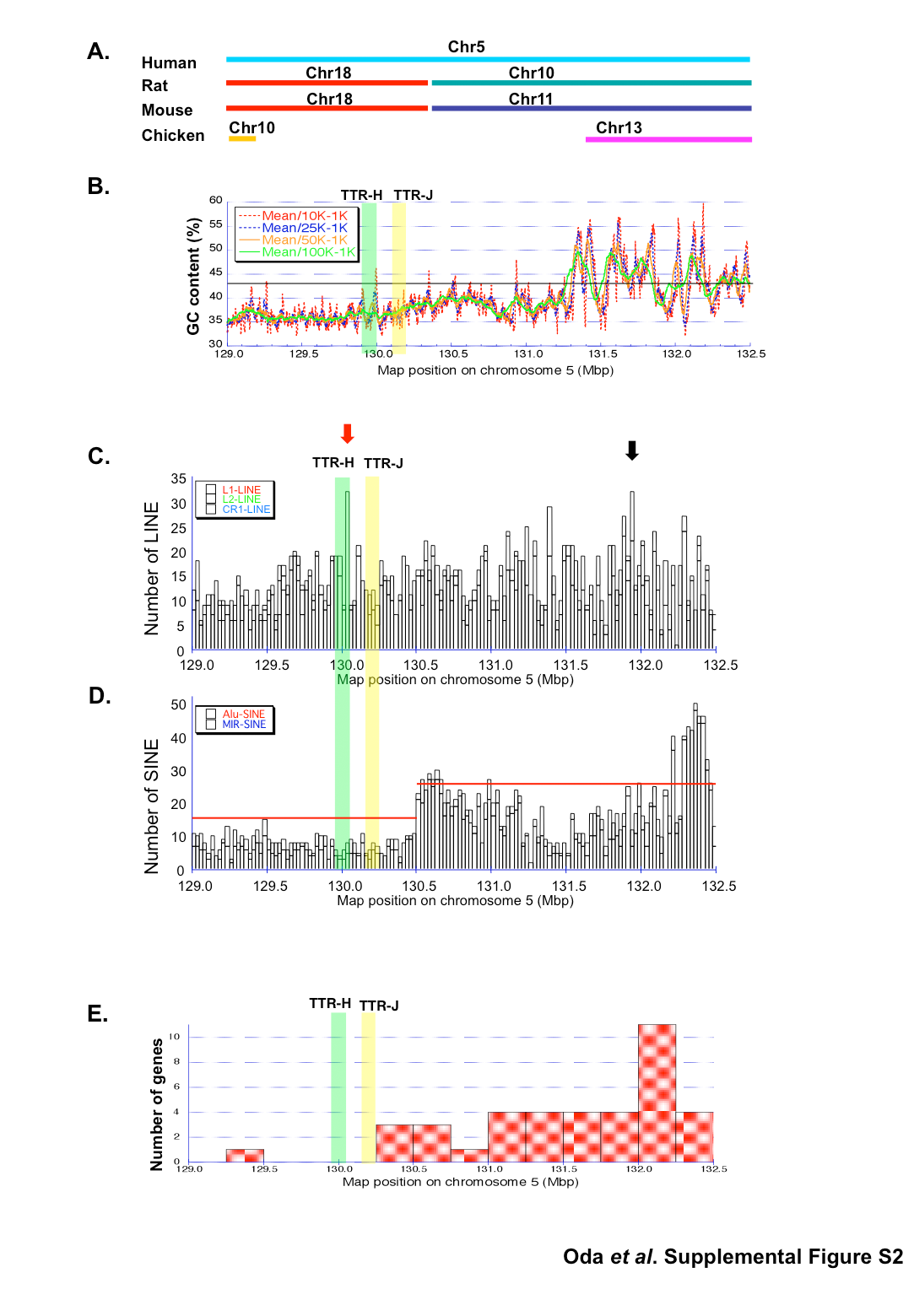

Supplement: Figure S2 — Genomic features of the human 5q23/31 region. A. Chromosomal synteny among human, rat, mouse and chicken corresponding to the human 5q23/31 region. B. Distribution of GC content (%). The GC contents were calculated by sliding window analyses across the genome region shown (sliding size, 1 kb; window size, 10–100 kb). The result indicates that early replicating segment is located in higher GC region (>43%). On the other hand, late replicating region as well as transition region are located in low GC region (<43%). C. Distribution of LINEs. A segment containing the most LINE1 was identified near the TTR (32 LINEs in the 25 kb segment, 130.025–130.05 Mb; red arrow). D. Distribution of SINEs. Average numbers of SINEs were calculated within the early- and late-replicating domains and are shown as horizontal red bars (19 SINEs/25 kb and 8 SINEs/25 kb, in early and late regions, respectively). Data were extracted from a RepeatMasker analysis (http://www.repeatmasker.org/) on the 129.0–132.5 Mb segment of the human chromosome 5. The numbers of LINE1 and SINEs were calculated by a sliding window analysis (sliding size: 25 kb, window size: 25 kb). E. Gene density. The total numbers of genes were calculated in non-overlapping windows of 250 kb each. The genes used in the analysis were taken from NCBI H. sapiens Genome (http://www.ncbi.nlm.nih.gov/). The gene density in the early replicating region is significantly higher than that in the late replicating region. In the vicinity of the IL-13/IL-4 gene loci, there are 11 genes in a 250 kb segment (130–132.25). Notably, no experimentally confirmed genes are present in the TTR. TTR-H and TTR-J are shown by green and yellow vertical bars, respectively. (TIF) [file pone.0042375.s002.tif]

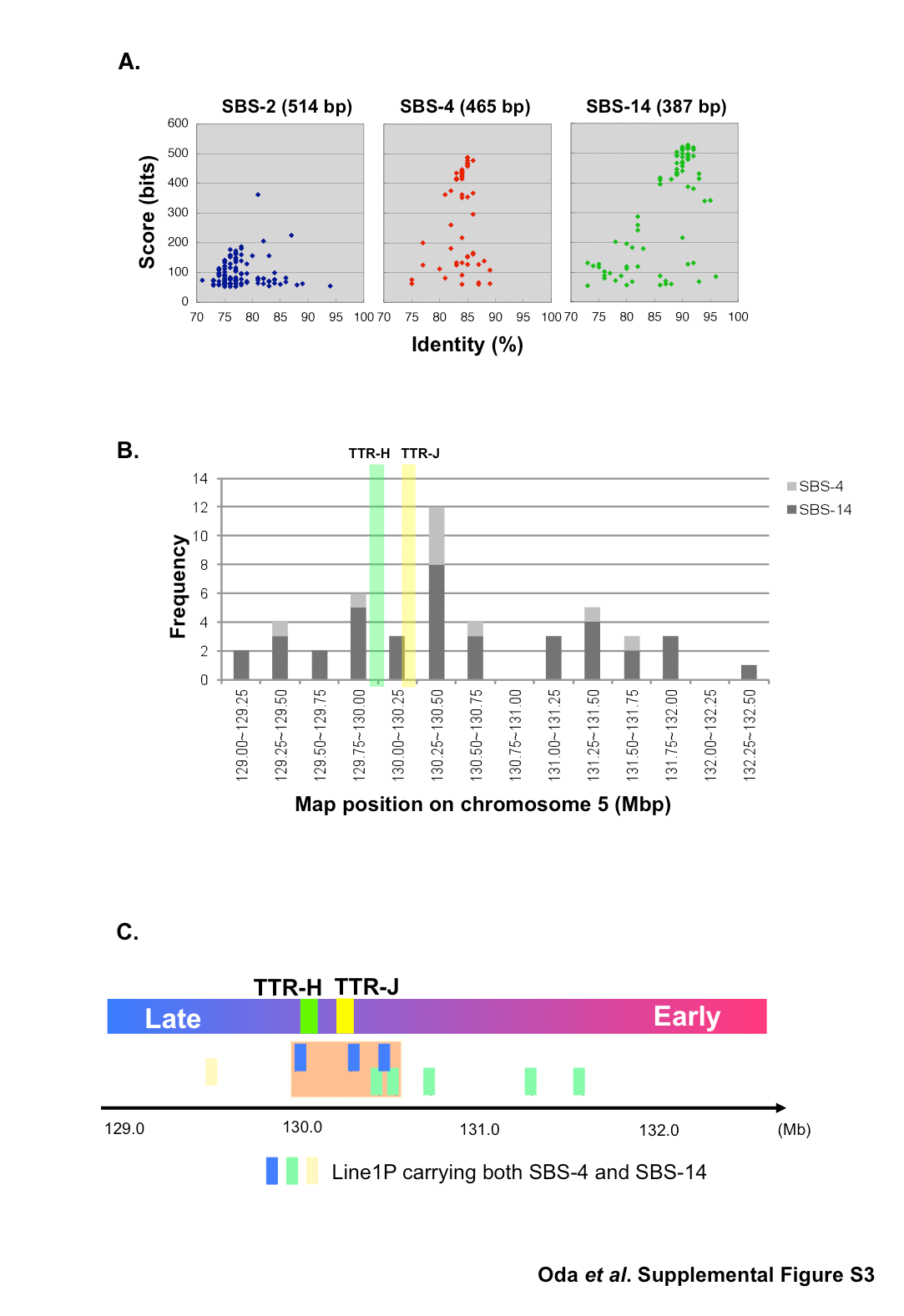

Supplement: Figure S3 — Distribution of potential SATB1 binding sequences (SBSs) at the human 5q23/31. A. Sequences similar to SBS-1∼16 on the 129–132.5Mb segment of human chromosome 5 were searched by using NCBI Blast (http://www.ncbi.nlm.nih.gov/BLAST/). Sequences similar to SBS-2 (514 bp), −4 (465 bp) and −14 (387 bp) were found, and “bit scores” were plotted against identity. B. Sequences with high identity (≧85%) and bit scores (≧400) as “highly similar” SBSs were selected (0 sequence for SBS-2, 9 sequences for SBS-4 and 39 sequences of SBS-14). Locations and numbers of these potential SBSs are shown on the human 5q23/31 3.5-Mb segment. C. Among them, nine pairs of SBS-4 and -14 that were found to be present on one Line1P (highlighted in yellow in Table S4) are indicated (blue, green and yellow small rectangles). Five of them were identified to be present close to the TTR (shown within the orange box). These findings lead us to suggest a possibility that the SBS clusters within and close to TTR may play a role in defining the replication timing boundary. Different colors for the rectangles indicate different orientations of SBS-4 and -14 on each Line1P. TTR-H and TTR-J are shown by green and yellow vertical bars, respectively. (TIF) [file pone.0042375.s003.tif]

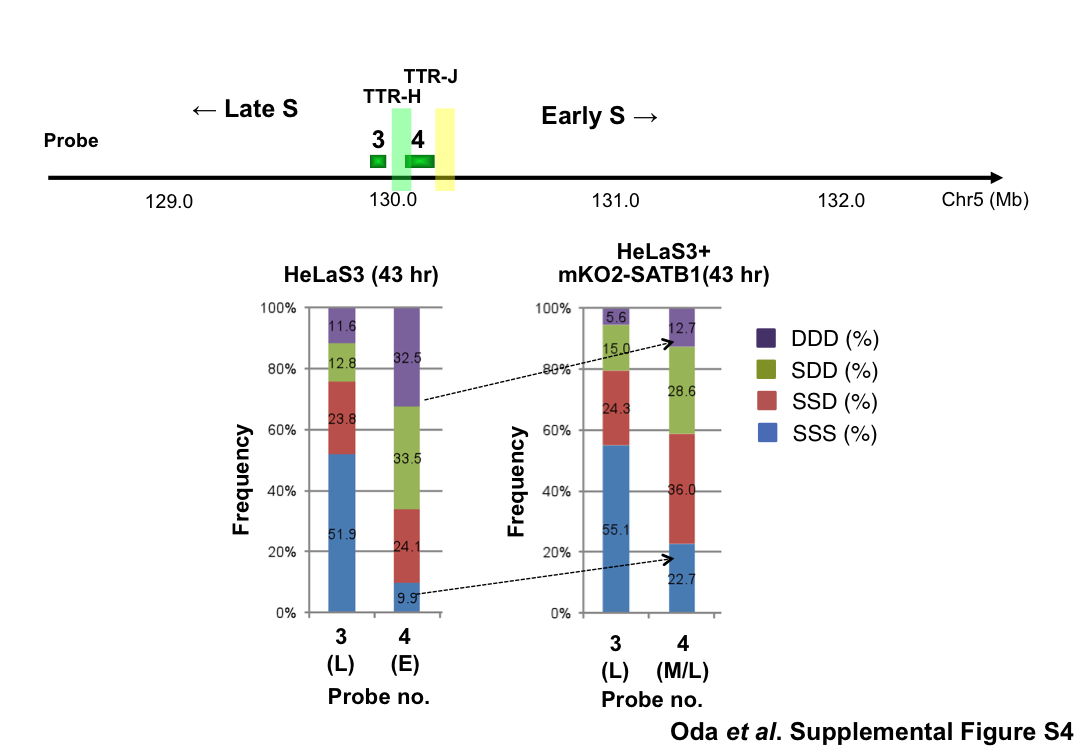

Supplement: Figure S4 — Effect of SATB1 expression on replication timing at TTR in HeLaS3 cells (with three chromosomes at the 5q locus). Replication timing of HeLaS3 and HeLaS3 expressing mKO2-SATB1 at 43 hr after transfection. We analyzed replication timing of HeLaS3 transfected with mKO2-SATB1 at 24 and 43 hr after transfection. Only the data at 43 hr are shown for cells non-transfected (left panel) or transfected with mKO2-SATB1 plasmid (right panel). At least 200 BrdU-positive nuclei (S-phase) containing three chromosomes at the 5q locus were counted for Probe 3 and 4. Replication timing in TTR (detected by Probe 4) changed from early (HeLaS3) to mid/late (HeLaS3 expressing mKO2-SATB1) (indicated by the arrows). (TIF) [file pone.0042375.s004.tif]

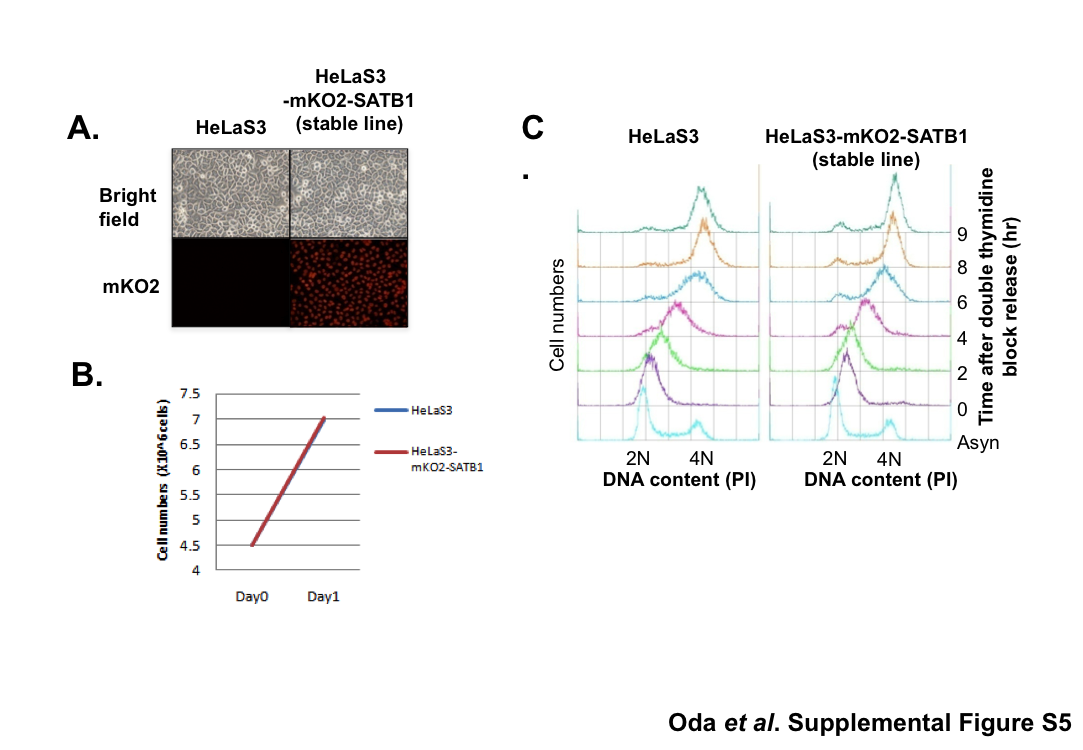

Supplement: Figure S5 — Cell growth and cell cycle of HeLaS3 and HeLaS3 cells stably expressing mKO2-SATB1. A. HeLaS3 and HeLaS3 cells stably expressing mKO2-SATB1 were observed by FSX100 (OLYMPUS). Red, mKO2 signal. B. Growth rate of HeLaS3 and stable mKO2-SATB1 HeLaS3. C. Stable mKO2-SATB1 HeLaS3 cells were synchronized at the G1/S boundary by double thymidine block, and then synchronously released into cell cycle and were collected at the times indicated (0, 2, 4, 6, 8 and 9 hrs). Collected cells were fixed in 70% ethanol, and DNA contents were analyzed by FACS. Growth rate and cell cycle distribution are not affected by expression of SATB1 in HeLaS3 cells. (TIF) [file pone.0042375.s005.tif]

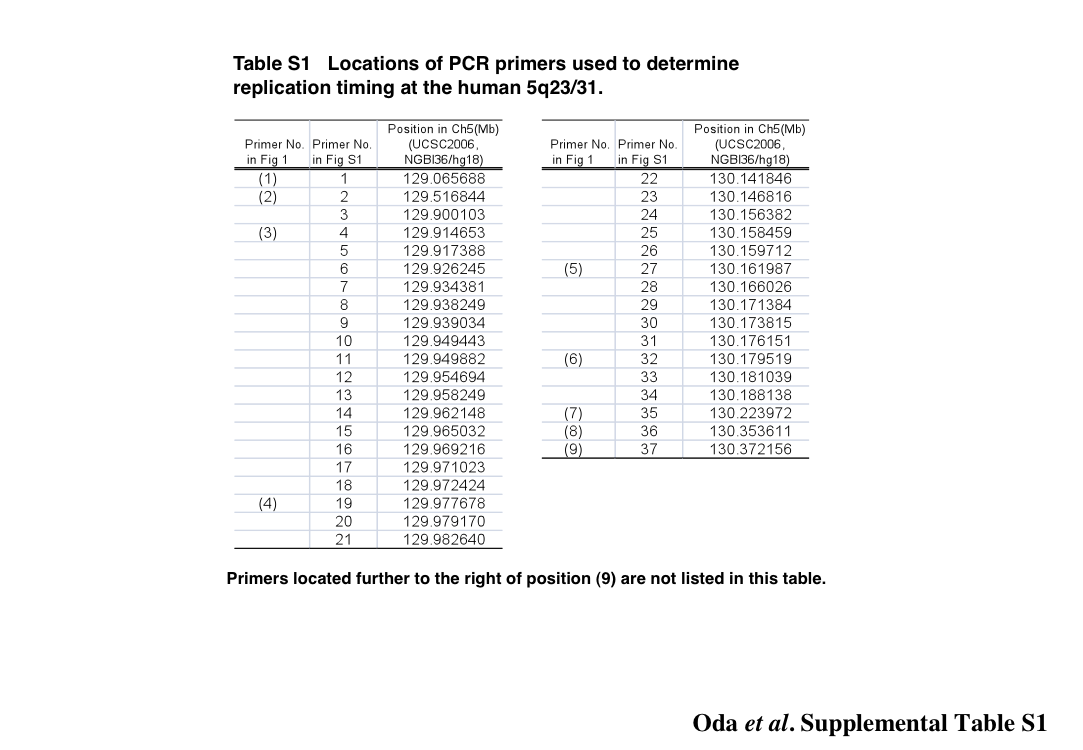

Supplement: Table S1 — Locations of PCR primers used to determine replication timing at the human 5q23/31. Primers located further to the right of position (9) are not listed in this table. (TIF) [file pone.0042375.s006.tif]

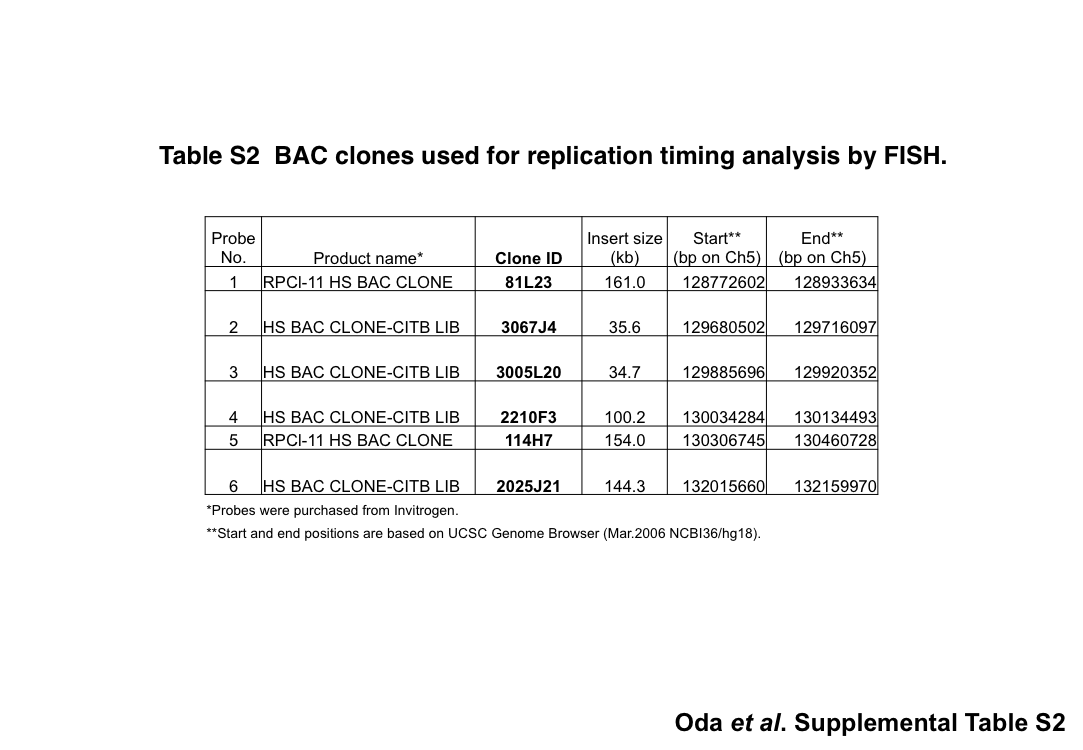

Supplement: Table S2 — BAC clones used for replication timing analyses by FISH. (TIF) [file pone.0042375.s007.tif]

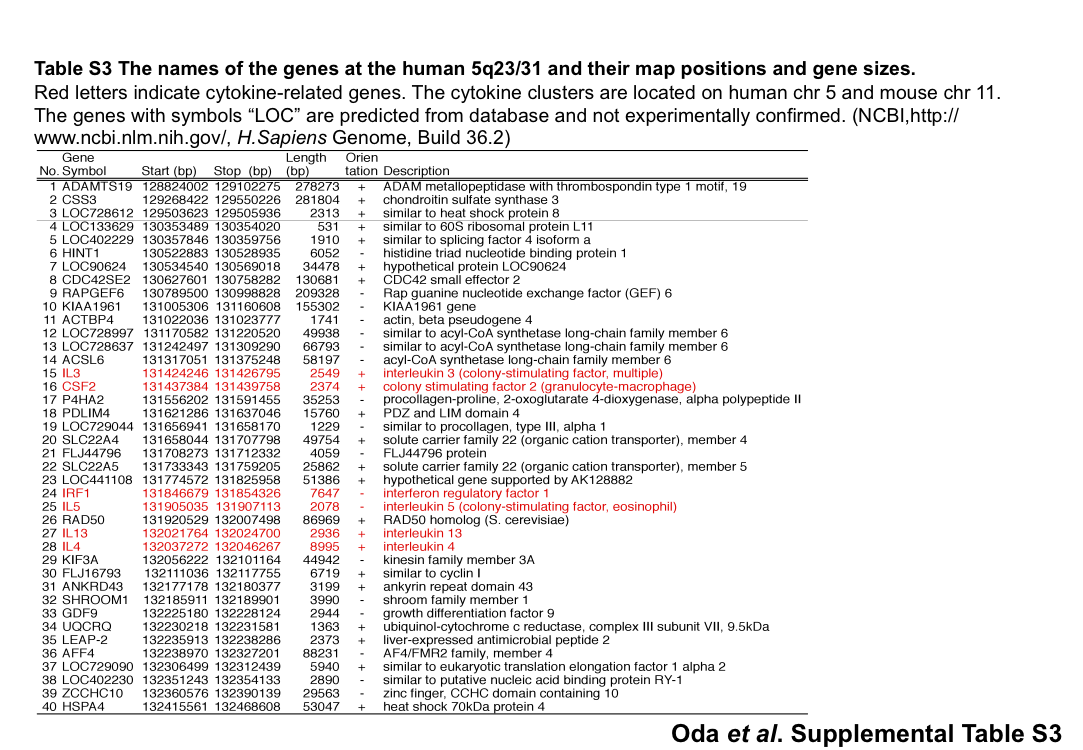

Supplement: Table S3 — The names of the genes at the human 5q23/31. and their map positions and gene sizes. (TIF) [file pone.0042375.s008.tif]

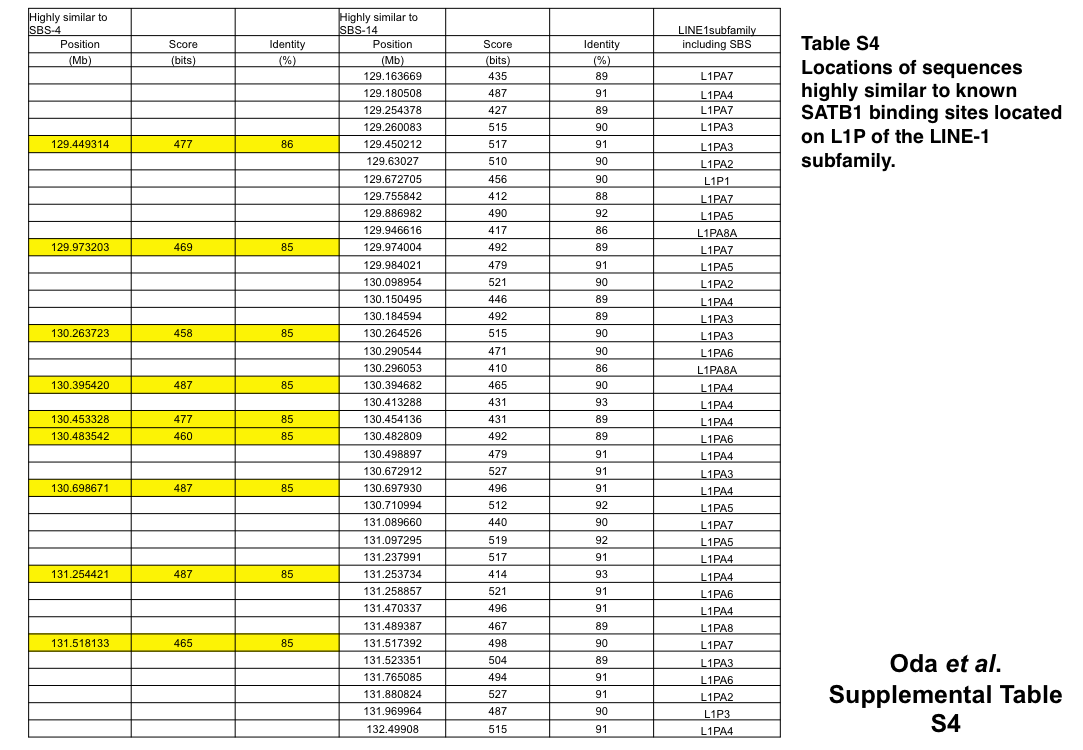

Supplement: Table S4 — Locations of sequences highly similar to known SATB1 binding sites located on L1P of the LINE-1 subfamily. Yellow columns indicate the Line1P sequences that carry both SBS-4 and 14, which are shown in Fig. S3C. (TIF) [file pone.0042375.s009.tif]

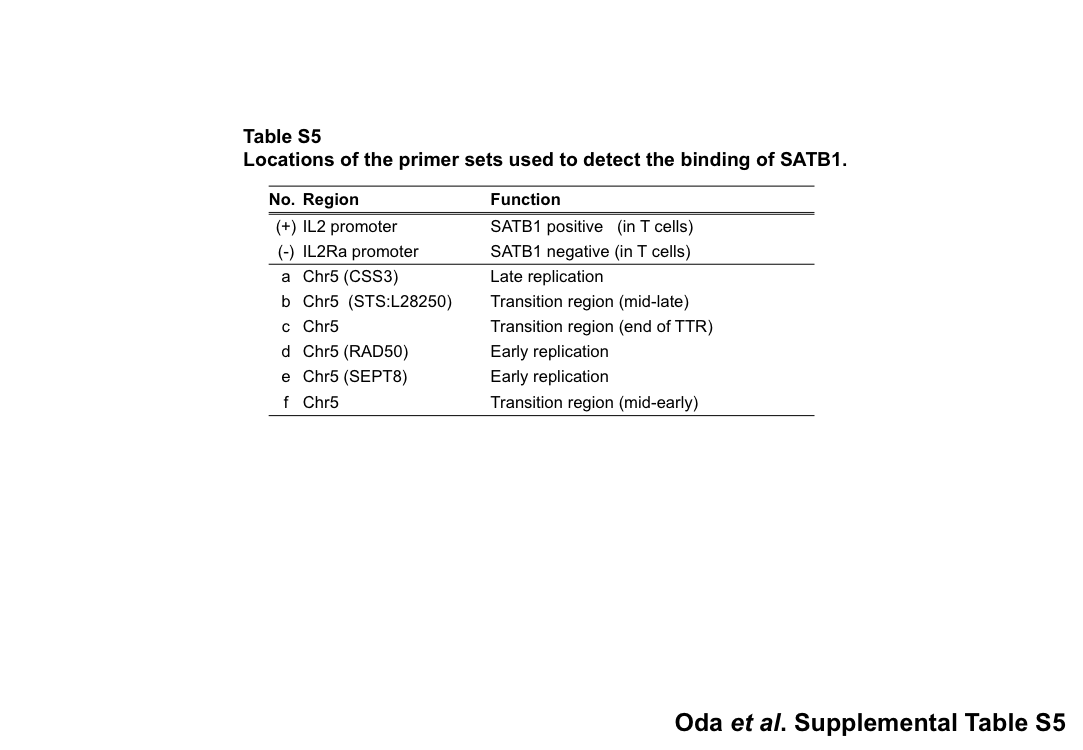

Supplement: Table S5 — Locations of the primer sets used for ChIP analyses of SATB1 binding and for copy number analyses. (TIF) [file pone.0042375.s010.tif]
